# Rimegepant orally disintegrating tablet 75 mg for acute treatment of migraine in adults from China: a subgroup analysis of a double-blind, randomized, placebo-controlled, phase 3 clinical trial

**DOI:** 10.1186/s10194-024-01731-4

**Published:** 2024-04-16

**Authors:** Shengyuan Yu, Aihong Guo, Zhen Wang, Jianguang Liu, Ge Tan, Qian Yang, Mingjie Zhang, Hasiyeti Yibulaiyin, Huisheng Chen, Yongbo Zhang, Robert Croop, Yanhui Sun, Yu Liu, Qian Zhao, Zhihong Lu

**Affiliations:** 1 Chinese PLA General Hospital, Beijing, China; 2 Xianyang Hospital, Yan’an University, Xianyang, China; 3 Changsha Central Hospital, Changsha, China; 4 Wuhan Third Hospital, Wuhan, China; 5 The First Affiliated Hospital of Chongqing Medical University, Chongqing, China; 6 Shaanxi Provincial Hospital, Xi’an, China; 7 The Second Affiliated Hospital of Xinjiang Medical University, Wulumuqi, Xinjiang Province China; 8 General Hospital of Northern Theater Command, District, Shenyang, Liaoning Province China; 9 Beijing Friendship Hospital, Capital Medical University, Beijing, China; 10 Biohaven Pharmaceuticals, New Haven, CT USA; 11 Pfizer (China) Research and Development Co., Ltd, Shanghai, China; 12 Pfizer Inc, Beijing, China; 13 Pfizer Inc, Chengdu, China

**Keywords:** Rimegepant, Migraine, Acute treatment, China, Clinical trial

## Abstract

**Background:**

Rimegepant orally disintegrating tablet (ODT), an oral small-molecule calcitonin gene-related peptide receptor antagonist, is indicated for acute and preventive treatment of migraine in the United States and other countries. Previously, a large clinical trial assessed the efficacy and safety of rimegepant ODT 75 mg for the acute treatment of migraine in adults living in China or South Korea. A post hoc subgroup analysis of this trial was performed to evaluate the efficacy and safety of rimegepant for acute treatment of migraine in adults living in China.

**Methods:**

Eligible participants were ≥ 18 years of age and had a ≥ 1-year history of migraine, with 2 to 8 attacks of moderate or severe pain intensity per month and < 15 headache days per month during the 3 months before screening. Participants self-administered rimegepant ODT 75 mg or matching placebo to treat a single migraine attack of moderate or severe pain intensity. The co-primary endpoints were pain freedom and freedom from the most bothersome symptom (MBS) at 2 h post-dose. Key secondary endpoints included pain relief at 2 h post-dose, ability to function normally at 2 h post-dose, use of rescue medication within 24 h post-dose, and sustained pain freedom from 2 to 24 h and 2 to 48 h post-dose. All *p* values were nominal. Safety was assessed via treatment-emergent adverse events (TEAEs), electrocardiograms, vital signs, and routine laboratory tests.

**Results:**

Overall, 1075 participants (rimegepant, *n* = 538; placebo, *n* = 537) were included in the subgroup analysis. Rimegepant was more effective than placebo for the co-primary endpoints of pain freedom (18.2% vs. 10.6%, *p* = 0.0004) and freedom from the MBS (48.0% vs. 31.8%, *p* <  0.0001), as well as all key secondary endpoints. The incidence of TEAEs was comparable between the rimegepant (15.2%) and placebo (16.4%) groups. No signal of drug-induced liver injury was observed, and no study drug-related serious TEAEs were reported in the rimegepant group.

**Conclusions:**

A single dose of rimegepant 75 mg rimegepant was effective for the acute treatment of migraine in adults living in China, with safety and tolerability similar to placebo.

**Trial registration:**

Clinicaltrials.gov NCT04574362 Date registered: 2020-10-05.

**Graphical Abstract:**

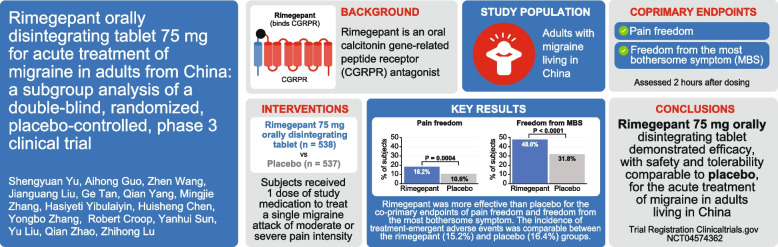

**Supplementary Information:**

The online version contains supplementary material, including a Plain Language Summary and Chinese versions of the manuscript and Plain Language Summary, available at 10.1186/s10194-024-01731-4.

## Background

Migraine is characterized by episodic, throbbing, moderate-to-severe headache pain that is typically accompanied by additional symptoms such as photophobia, phonophobia, and nausea or vomiting [1]. Migraine is among the most prevalent and disabling disorders globally and can negatively impact patient activity, social interaction, workplace productivity, and overall quality of life [2–9]. Despite being a global concern, the quality of migraine care varies across countries due to discrepancies in provider education and resources, access to treatment, cost of treatment, and other socioeconomic factors [10, 11].

Migraine affects approximately 151.6 million people in China [3]. However, migraine is generally under-recognized in China, with misdiagnosis a key concern [12, 13]. Roughly half of people with migraine in China do not seek medical attention and treat migraine attacks with over-the-counter medication such as aspirin and non-steroidal anti-inflammatory drugs (NSAIDs) [14, 15]. Those seeking medical consultation will typically be treated by primary care physicians, rather than neurologists/specialists, and there are few headache centers in China [15, 16]. Although migraine treatment guidelines in China are generally consistent with those of Western countries, the concept of migraine prevention is not well adopted in China and use of preventive medications is low, even in headache clinics [12, 13, 16]. This may be related to the overall low diagnosis rate of migraine in China and that most prophylactic treatments are not officially approved (or reimbursed) for an indication of preventive treatment of migraine in China.

Guidelines for the acute treatment of migraine in China recommend NSAIDs, acetaminophen, caffeinated analgesic compounds, triptans, lasmiditan, rimegepant, and ubrogepant [17]. Based on a retrospective analysis of medical insurance claims among adult participants with migraine (*n* = 10652) in China, only 26.4% (*n* = 2813) of participants received an acute medication prescribed for migraine or pain relief [16]. Among these 2813 participants, the most commonly prescribed medications were non-aspirin NSAIDs (68.8%), followed by aspirin (8.0%), opioids (7.1%), ergot alkaloids (6.1%), and acetaminophen (4.3%) [16]. Notably, only 3.3% of participants were prescribed triptans, which are recommended for acute treatment of migraine in guidelines from China (and other countries) and are commonly prescribed acute treatments in the United States and the European Union [16, 18–24]. Unlike Western countries, Chinese patent and herbal medicines are also commonly used to treat migraine in China [16, 25].

Some of the acute therapies described above have limitations. Triptans and NSAIDs, for example, are contraindicated in participants with cardiovascular disease and/or their use is cautioned in those with cardiovascular risk factors [26, 27]. Additionally, NSAIDS, triptans, ergot alkaloids, and combination analgesics are associated with a risk of medication overuse headache (MOH) [1, 28]. MOH is a concern in China as it is has a significant impact on patient quality of life, is often undiagnosed, or is erroneously considered as worsening of migraine [29]. In a retrospective study of 1453 adults with migraine at a headache treatment center in China, 6.5% (*n* = 240) met diagnostic criteria for MOH [29]. Finally, many patients respond poorly to these acute therapies; studies in China estimate that more than 40% of patients have insufficient response to acute treatment [8, 30]. Insufficient response can lead to dissatisfaction with treatment and is a risk factor for transformation of episodic migraine to chronic migraine [31]. Thus, there is a clear unmet need in China for safe and effective acute treatments for migraine [8, 30, 32].

A key role for calcitonin gene-related peptide (CGRP) has been established in the pathophysiology of migraine, with several agents that target CGRP signaling approved for migraine treatment in the United States and European Union [33]. Despite guidelines in China recommending the use of CGRP receptor antagonists for acute treatment of migraine, ubrogepant is not currently approved and rimegepant was only recently approved in China (January 2024) and not yet eligible for reimbursement [17, 34]. Rimegepant is indicated for acute treatment of migraine and for preventive treatment of episodic migraine in the United States, European Union, and United Kingdom [35, 36]. The efficacy and safety of rimegepant for acute treatment of migraine was initially established in three pivotal, randomized, placebo-controlled, Phase 3 clinical trials and an open-label, long-term (1 year) Phase 2/3 safety trial conducted in the United States [37–40]. A subsequent randomized, placebo-controlled, Phase 3 trial with the rimegepant 75 mg orally disintegrating tablet (ODT) formulation for acute treatment of migraine demonstrated efficacy and safety in adults living in China or South Korea (NCT04574362), and was the first clinical trial of rimegepant for the acute treatment of migraine conducted outside the United States [41]. In Chinese and Korean adults, rimegepant was superior to placebo on all primary and key secondary efficacy endpoints, with safety and tolerability comparable to placebo [41].

It is possible that response to rimegepant could vary among Korean and Chinese participants due to differences in demographics, approach/access to migraine treatment, and other social or cultural factors. Additionally, there is a lack of data on the use of rimegepant for acute treatment of migraine specifically in Chinese adults. Therefore, we conducted a subgroup analysis (based on previous trial NCT04574362 in Korean and Chinese participants) to assess the efficacy and safety of rimegepant ODT 75 mg in adults with migraine who live in China.

## Methods

### Study design and eligibility criteria

**Fig. 1:**
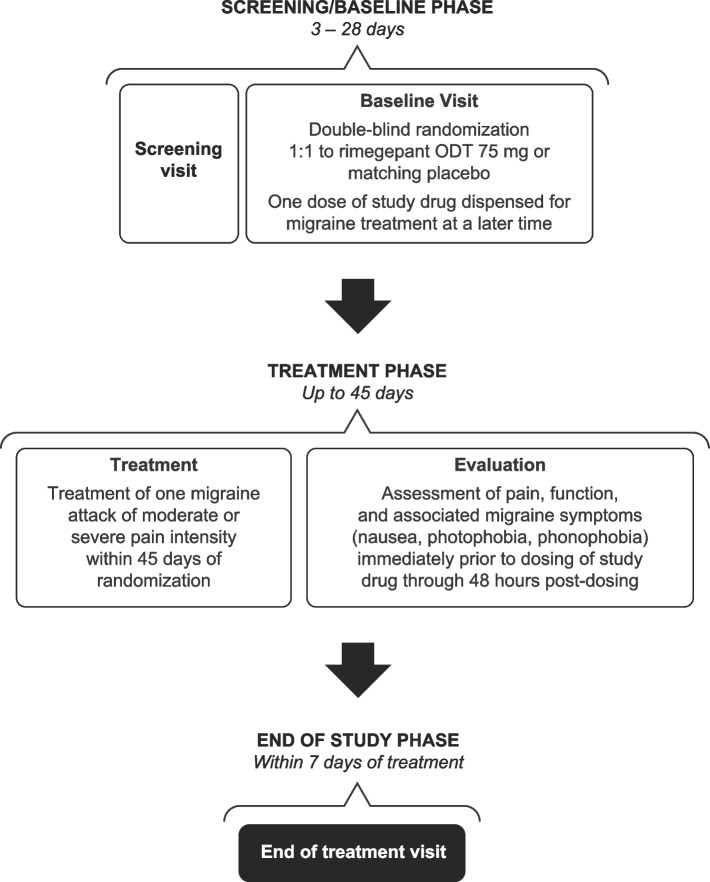
Study design. ODT, orally disintegrating tablet

Full study methods and details on ethical oversight have been published previously [41]. Briefly, the study (NCT04574362) was comprised of a 3 to 28-day screening period, an acute treatment phase lasting up to 45 days or until the participant had a migraine attack of moderate or severe pain intensity, and an end of treatment visit within 7 days after the administration of study medication (Fig. 1).

Key inclusion criteria included: age ≥ 18 years; a ≥ 1-year history of headache consistent with a diagnosis of migraine (with or without aura) according to the International Classification of Headache Disorders 3rd Edition (beta version) [42]; and 2 to 8 attacks of moderate or severe pain intensity per month, with migraine attacks that last an average of 4 to 72 h if untreated, and < 15 headache days per month during the 3 months before screening. Participants with a history of migraine with brainstem aura or hemiplegic migraine were excluded. Participants with contraindications to triptans were eligible if they met all other entry criteria. Participants on preventive migraine medication were permitted to remain on preventive therapy provided dosing was stable for ≥3 months prior to screening. Participants who have previously participated in studies of investigational CGRP-antagonists (small molecule or biologic) or have been prescribed CGRP-antibodies within the last 6 months were excluded.

### Treatment

Participants meeting eligibility criteria were randomized, in a double-blind manner via the sponsor’s Interactive Web Response System, in a 1:1 ratio to either rimegepant ODT 75 mg or matching placebo treatment. Randomization was stratified by country (China and South Korea) and use of preventive medication (yes vs. no). Upon randomization, participants were provided a single dose of study medication to treat (via self-administration) a migraine attack of moderate or severe pain intensity within 45 days.

All other headache medications were prohibited for 2 h post-dosing of study medication. Participants who did not experience pain relief 2 h post-dose (or if the migraine that was relieved at 2 h returned to a moderate or severe pain intensity between 2 and 48 h) were permitted to use the following rescue medications: aspirin, ibuprofen, acetaminophen up to 1000 mg/day, NSAIDs, antiemetics, or baclofen. If needed, participants could take prescribed standard-of-care migraine treatments 48 h after dosing of study medication. Use of rescue medication was recorded by the participant in a paper diary.

### Efficacy assessments

Participants recorded efficacy data in an Electronic Diary, including the time of headache onset, pain intensity, the presence or absence of associated migraine symptoms, and ratings of functional disability. A 4-point numeric rating scale was used to rate pain severity (0 = none, 1 = mild, 2 = moderate, 3 = severe) and functional disability (0 = normal, 1 = mildly impaired, 2 = severely impaired, 3 = requires bedrest). The presence of associated migraine symptoms (nausea, photophobia, and phonophobia) was assessed using a binary scale (0 = absent, 1 = present) and participants were asked to identify, prior to dosing, their most bothersome symptom (MBS), selected among nausea, photophobia, and phonophobia. Assessments of pain severity, presence of associated migraine symptoms, and functional disability were performed immediately prior to dosing and at 15 min, 30 min, 45 min, 60 min, 90 min, 2 h, 3 h, 4 h, 6 h, 8 h, 24 h, and 48 h after dosing.

Pain freedom and freedom from the MBS were defined as a score of 0 on the respective numeric rating scales. Pain relief was defined as a score of 0 or 1 on the pain rating scale.

### Efficacy endpoints

The co-primary endpoints were the proportion of participants with pain freedom at 2 h post-dose and the proportion of participants with freedom from the MBS at 2 h post-dose. Key secondary endpoints included the proportion of participants with pain relief at 2 hours post-dose, the proportion of participants with normal function at 2 h post-dose (among participants with any level of disability prior to taking study medication), the proportion of participants using rescue medication within 24 h post-dose, the proportion of participants with sustained pain freedom from 2 to 24 h post-dose, and the proportion of participants with sustained pain freedom from 2 to 48 h post-dose.

Other secondary or exploratory endpoints included the proportion of participants with pain freedom, MBS freedom, pain relief, and normal function at 15 min, 30 min, 45 min, 60 min, 90 min, 3 h, 4 h, 6 h, 8 h, 24 h, and 48 h post-dose. The proportion of participants who had pain relapse (pain severity score of 1, 2, or 3) at any point from 2 to 48 h post-dose was also assessed among those with pain freedom at 2 h post-dose.

### Safety assessments

Safety was assessed via treatment-emergent adverse events (TEAEs), electrocardiograms (ECG), vital signs, physical examinations, and routine laboratory tests. TEAEs were defined as those with onset date on or after the study medication date. TEAEs were summarized descriptively, with severity and relationship to study treatment determined by site investigators. TEAEs were coded using Medical Dictionary for Regulatory Activities v23.0.

### Statistical considerations

All analyses were conducted in the subgroup of Chinese participants. Analysis of the co-primary endpoint and key secondary efficacy endpoints in this subgroup were pre-specified in the statistical analysis plan, whereas analyses of the other secondary efficacy endpoints in this subgroup were post hoc. Efficacy was analyzed in all randomized Chinese participants who received study medication, had a migraine attack of moderate or severe pain intensity at the time of dosing, and provided at least one efficacy datapoint after receiving study treatment. Safety was assessed in all Chinese participants who received study treatment.

Rimegepant was compared with placebo at a two-sided alpha level of 0.05 for the co-primary and secondary efficacy endpoints using Cochran-Mantel-Haenszel tests stratified by use of preventive migraine medication. Statistical analyses in the Chinese subgroup did not control for Type 1 error and all *p* values were nominal. For the endpoints of pain freedom, MBS freedom, pain relief, and normal function, participant data were imputed as failures if there were missing data at the time point being assessed or participants used rescue medication at or before the time point being assessed (participants not reporting a MBS at migraine onset were also imputes as failures in the MBS freedom analysis). For the endpoint of sustained pain freedom from 2 to 24 h, participant data were imputed as failures if there were missing data at 2 or 24 h, had missing data at > 1 other time point (3, 4, 6, or 8 h), or used rescue medication at or before 24 h. For the endpoint of sustained pain freedom from 2 to 48 h, participant data were imputed as failures if there were missing data at 2, 24, or 48 h, had missing data at > 1 other time point (3, 4, 6, and 8 h), or used rescue medication at or before 48 h. For the endpoint of pain relapse, participant data were imputed as failures (i.e., had pain relapse) if participants had missing data at 24 or 48 h, had missing data at > 1 time point, or if they used rescue medication from 2 to 48 h. All statistical analyses were performed using SAS version 9.4 (Cary, NC, USA).

## Results

### Participants

A total of 1075 Chinese participants (rimegepant, *n* = 538; placebo, *n* = 537) received study treatment. Demographics and clinical characteristics for all treated Chinese participants are shown in Table 1. These participants had a median age of 35 (range, 18–71) years and a majority (79.0%) were female. The median (range) number of moderate or severe migraine attacks per month was 3 (2–8), and nausea was the most frequently reported historical MBS (52.0%). Preventive migraine medications were used by 3.5% of the Chinese study population.

**Table 1:** Demographics and clinical characteristics in all Chinese participants receiving study treatment

Demographic	Rimegepant 75 mg *n* = 538	Placebo *n* = 537	Overall *N* = 1075
Age, years			
Mean (SD)	37.3 (10.2)	36.7 (10.4)	37.0 (10.3)
Median (range)	36 (19–71)	35 (18–70)	35 (18–71)
Sex, n (%)			
Female	412 (76.6)	437 (81.4)	849 (79.0)
Male	126 (23.4)	100 (18.6)	226 (21.0)
Body mass index^a^, kg/m^2^			
Mean (SD)	22.9 (3.4)	23.1 (3.4)	23.0 (3.4)
Median (range)	22.5 (15.6–47.0)	22.8 (15.5–35.8)	22.7 (15.5–47.0)
Primary migraine type, n (%)			
Without aura	476 (88.5)	476 (88.6)	952 (88.6)
With aura	62 (11.5)	61 (11.4)	123 (11.4)
Age at disease onset^b^, years			
Mean (SD)	27.1 (9.2)	26.2 (8.6)	26.7 (8.9)
Median (range)	27 (3–49)	26 (6–49)	26 (3–49)
Average duration of untreated attacks, hours			
Mean (SD)	18.0 (14.7)	18.4 (14.7)	18.2 (14.6)
Median (range)	12 (4–72)	12 (4–72)	12 (4–72)
Number of attacks with moderate or severe pain intensity per month			
Mean (SD)	3.5 (1.3)	3.4 (1.2)	3.5 (1.2)
Median (range)	3 (2–8)	3 (2–8)	3 (2–8)
Historically most bothersome symptom, n (%)			
Nausea	281 (52.2)	278 (51.8)	559 (52.0)
Phonophobia	155 (28.8)	157 (29.2)	312 (29.0)
Photophobia	102 (19.0)	101 (18.8)	203 (18.9)
Missing	0	1 (0.2)	1 (0.1)
Took preventive migraine treatment previously, n (%)	21 (3.9)	17 (3.2)	38 (3.5)

*SD* Standard deviation

^a^Participant number = 537 for rimegepant, 537 for placebo, and 1074 for overall

^b^Participant number = 532 for rimegepant, 532 for placebo, and 1064 for overall

### Efficacy

At 2 h post-dose, rimegepant was more effective than placebo on the co-primary endpoints of freedom from pain (18.2% vs. 10.6%; risk difference = 7.6; *p* = 0.0004) and freedom from the MBS (48.0% vs. 31.8%; risk difference = 16.2; *p* <  0.0001) (Table 2).

**Table 2:** Summary of co-primary and key secondary endpoints^a^

Endpoint	Rimegepant 75 mg *n* = 537	Placebo *n* = 537	Risk Difference^b^ (95% CI)	*p* value^c^
**Co-Primary**				
Pain freedom at 2 h post-dose	98 (18.2%)	57 (10.6%)	7.6 (3.5, 11.8)	0.0004
MBS freedom at 2 h post-dose	258 (48.0%)	171 (31.8%)	16.2 (10.4, 22.0)	< 0.0001
**Key Secondary**				
Pain relief at 2 h post-dose	351 (65.4%)	256 (47.7%)	17.8 (12.0, 23.7)	< 0.0001
Normal function at 2 h post-dose ^d^	176 (38.5%)	110 (23.8%)	14.7 (8.8, 20.6)	< 0.0001
Rescue Medication use within 24 h post-dose	28 (5.2%)	75 (14.0%)	−8.9 (−12.4, −5.4)	< 0.0001
Sustained pain freedom from 2 to 24 h post-dose	82 (15.3%)	43 (8.0%)	7.2 (3.4, 11.1)	0.0002
Sustained pain freedom from 2 to 48 h post-dose	78 (14.5%)	39 (7.3%)	7.2 (3.5, 10.9)	0.0001

*MBS* Most bothersome symptom

^a^Includes all randomized Chinese participants who took study treatment, had a migraine of moderate or severe intensity at the time of treatment, and provided at least one post-treatment efficacy data point. See methods section for details on handling of missing data and definition of failures

^b^Rimegepant vs. placebo, calculated from Mantel–Haenszel test stratified by preventive migraine medication use

^c^Rimegepant vs. placebo, calculated from Cochran-Mantel-Haenszel test stratified by preventive migraine medication use. All *p* values are nominal

^d^Among participants with functional disability at time of dosing (rimegepant, *n* = 457, placebo, *n* = 462)

Rimegepant was also more effective than placebo on all key secondary endpoints (Table 2), including pain relief at 2 h post-dose (65.4% vs. 47.7%; *p* <  0.0001), normal function at 2 h post-dose (38.5% vs. 23.8%; *p* <  0.0001), the use of rescue medication within 24 h post-dose (5.2% vs. 14.0%; *p* <  0.0001), sustained pain freedom from 2 to 24 h post-dose (15.3% vs. 8.0%; *p* = 0.0002), and sustained pain freedom from 2 to 48 h post-dose (14.5% vs. 7.3%; *p* = 0.0001).

**Fig. 2:**
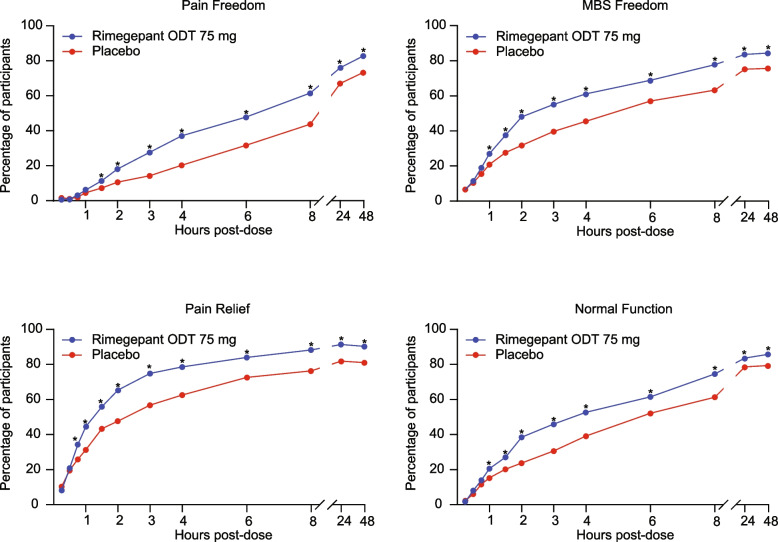
Time-course of the proportion of participants with pain freedom, MBS freedom, pain relief, and normal function from 15 minutes to 48 hours post-dose. Assessments were made at 15 min, 30 min, 45 min, 1 h, 1.5 h, 2 h, 3 h, 4 h, 6 h, 8 h, 24 h, and 48 h post-dose. Participants with missing data at the time point being assessed or who used rescue medication at or before the time point being assessed were imputed as failures (participants not reporting a MBS at migraine onset were imputed as failures in the MBS freedom analysis). *Nominal *p* <  0.05; based on the risk difference (rimegepant ODT vs. placebo). MBS, most bothersome symptom; ODT, orally disintegrating tablet

The time-course of pain freedom, MBS freedom, pain relief, and normal function from 15 min to 48 h post-dose is shown in Fig. 2 and Supplementary Table 1. Rimegepant demonstrated improvements over placebo (*p* <  0.05) as early as 90 min post-dose for pain freedom, 60 min for MBS freedom, 45 min for pain relief, and 60 min for normal function. For each of these endpoints, improvements over placebo (*p* <  0.05) were maintained at all subsequent time points through 48 h post-dose. Among participants with pain freedom at 2 h post-dose, 20.4% had pain relapse up to 48 h in the rimegepant group compared with 31.6% in the placebo group (*p* = 0.1382).

### Safety

The overall rate of TEAEs was 15.2% in the rimegepant group and 16.4% in the placebo group (Table 3). Rates of the most frequently reported TEAEs (those reported in ≥0.5% of rimegepant-treated participants) were comparable between treatment groups. Of these TEAEs, only protein urine present (rimegepant, 1.5%; placebo, 1.3%) and nausea (rimegepant, 1.1%; placebo, 2.6%) were reported in ≥1% of the rimegepant group.

**Table 3:** Summary of treatment-emergent adverse events in all treated Chinese participants

TEAE, *n* (%)	Rimegepant 75 mg *n* = 538	Placebo *n* = 537
Any TEAE	82 (15.2)	88 (16.4)
Serious TEAE	1 (0.2)^a^	2 (0.4)
Most common TEAEs^b^		
Protein urine present	8 (1.5)	7 (1.3)
Nausea	6 (1.1)	14 (2.6)
Urinary tract infection	5 (0.9)	8 (1.5)
Blood creatine phosphokinase increased	5 (0.9)	3 (0.6)
Proteinuria	4 (0.7)	1 (0.2)
Photophobia	4 (0.7)	3 (0.6)
Upper respiratory tract infection	3 (0.6)	4 (0.7)
Blood urine present	3 (0.6)	2 (0.4)
Blood glucose increased	3 (0.6)	1 (0.2)
Urine leukocyte esterase positive	3 (0.6)	1 (0.2)
**Study drug-related**		
Any TEAE	45 (8.4)	43 (8.0)
Serious TEAE	0	1 (0.2)

MedDRA, *ODT* Orally disintegrating tablet, *TEAE* Treatment emergent adverse event

^a^This serious TEAE was classified using the MedDRA (version 23.0) preferred term “infection”

^b^TEAEs occurring in ≥0.5% of participants in the rimegepant ODT 75 mg group

Serious TEAEs occurred in 1 (0.2%) participant in the rimegepant group and 2 (0.4%) in the placebo group. Study drug-related serious TEAEs occurred in no participants in the rimegepant group and 1 (0.2%) in the placebo group. The overall rate of study drug–related TEAEs was 8.4% with rimegepant treatment and 8.0% with placebo.

No deaths were reported and no clinically meaningful changes in ECGs, vital signs, physical examination results, or routine laboratory tests were observed in either treatment group. There were no participants with alanine transaminase or aspartate aminotransferase concentrations >3x the upper limit of normal and concurrent concentrations of total bilirubin >2x the upper limit of normal.

## Discussion

A previous randomized, placebo-controlled, Phase 3 trial demonstrated efficacy and safety of rimegepant ODT 75 mg for the acute treatment of migraine in adults from China or South Korea and confirmed that findings from US-based populations can be generalized to Chinese and Korean populations [41]. However, there is a lack of data on the use of rimegepant for acute treatment of migraine specifically in Chinese adults. Therefore, we conducted a subgroup analysis, based on the previous trial in Korean and Chinese participants, to assess the efficacy and safety of rimegepant ODT 75 mg in adults with migraine living in China. In this subgroup of Chinese adults, rimegepant was more effective than placebo on all co-primary and key secondary efficacy endpoints and demonstrated a TEAE profile comparable to placebo. These subgroup results mirror findings observed in the overall study population [41] and confirm rimegepant is effective as an acute treatment of migraine in Chinese adults.

Specifically, in Chinese participants in this study, rimegepant was associated with improvements over placebo for the co-primary efficacy endpoints of freedom from pain and freedom from the MBS at 2 h post-dose. Rimegepant also demonstrated improvement over placebo on the endpoints of pain relief and return to normal function at 2 h post-dose. Additionally, fewer participants in the rimegepant group required rescue medication through 24 h post-dose compared with the placebo group. Improvements over placebo were observed at 45 min post-dose for the endpoint of pain relief, 60 min for the endpoints of normal function and MBS freedom, and 90 min for pain freedom. These improvements for pain freedom, MBS freedom, pain relief, and normal function were observed at all subsequent assessments through 48 h post-dose, which suggests a sustained effect of rimegepant in many participants. This is further supported by the observation that the rimegepant group had a greater proportion of participants with sustained pain freedom from 2 to 24 h and from 2 to 48 h post-dose compared with the placebo group. Overall, these findings in Chinese participants are consistent with findings from previous US-based studies of rimegepant for the acute treatment of migraine, where improvements over placebo were demonstrated for pain freedom, MBS freedom, pain relief, and normal function at 2 h post-dose, for use of rescue medication through 24 h post-dose, and for sustained pain freedom from 2 to 24 and 2 to 48 h post-dose [37, 38, 40].

The safety findings observed in Chinese participants are also consistent with previous US-based trials of rimegepant since rimegepant demonstrated an overall safety profile comparable to placebo [37, 38, 40]. In the subgroup of Chinese participants, no treatment-related serious TEAEs were reported in the rimegepant group, a majority of TEAEs were mild or moderate in severity, most TEAEs resolved without treatment, TEAEs occurred at a similar rate in the rimegepant and placebo groups, there were no clinically meaningful changes in ECGs or routine laboratory tests observed, and there was no signal of hepatotoxicity.

The evidence for efficacy and a favorable safety profile of rimegepant demonstrated in this subgroup analysis suggests rimegepant may help address an unmet need for safe and effective acute treatment of migraine in China. Guidelines for acute treatment of migraine in China recommend NSAIDs, which are the most commonly prescribed acute medication for migraine in China [43]. However, NSAIDs may not be effective against migraine attacks of severe pain intensity [18, 44] and long-term use of these agents is associated with gastrointestinal bleeding, cardiovascular thrombotic events, and renal damage [45–47]. As a result, use of these agents is cautioned in patients with or at risk for gastrointestinal events, cardiovascular disease, or renal impairment [27]. Triptans are also recommended by Chinese treatment guidelines, but are not commonly utilized in China [16, 43]. This may be due to a variety of factors, including cost, access, and that triptans are contraindicated for patients with cardiovascular disease due to their vasoconstrictive properties [26]. NSAIDs, triptans, and other acute migraine treatments also carry a risk of MOH [28]. In contrast, rimegepant, like other gepant medications (i.e., zavegepant, ubrogepant) is not associated with MOH [48]. For example, scheduled every other day dosing of rimegepant for 12 weeks for preventive treatment of migraine resulted in significant reduction of monthly migraine days compared to placebo without evidence of MOH [49]. Additionally, real-word evidence shows that use of rimegepant for migraine therapy reduces both the point prevalence of MOH and the requirement for certain medications that can cause MOH, including barbiturates and opioids [50–52].

Though the exact mechanism underlying MOH is not known, it is thought to involve changes in descending pain modulation, sensitization of nociceptors, other structural and function alterations in the nervous system, and, possibly, biobehavioral factors [53, 54]. Repeated administration of gepants has not been associated with sensory changes suggestive of MOH in preclinical models of medication overuse [55, 56].

Although rimegepant demonstrated efficacy and a favorable safety profile for acute treatment of migraine in this study, the study was designed to assess the effects of a single dose of rimegepant and, as a result, does not allow for conclusions on the efficacy or safety of repeated long-term use of rimegepant in Chinese adults. Additional ongoing studies (NCT05371652, NCT05810038) will assess the long-term use of rimegepant in Chinese populations. In addition, statistical analyses in the current subgroup analysis of Chinese participants did not control for type 1 error and all *p* values were nominal.

Overall, this study demonstrated that a single dose of rimegepant ODT 75 mg is effective and well tolerated for acute treatment of migraine in adults living in China. These results indicate that use of rimegepant could, potentially, help address an unmet need for safe and effective acute treatments of migraine in the People’s Republic of China. This conclusion is supported by guidelines on the diagnosis and treatment of migraine in China, which strongly recommends rimegepant as acute therapy based on high level evidence of efficacy and favorable safety profile established across multiple randomized, placebo-controlled clinical trials [17].

### Supplementary Information

**Additional file 1: Supplementary Table 1.**

**Additional file 2.** English plain langauge summary.

**Additional file 3.** Chinese plain langauge summary.

**Additional file 4.** Chinese full manuscript.

## Data Availability

Upon request, and subject to review, Pfizer will provide the data that support the findings of this study. Pursuant to certain criteria, conditions, and exceptions, Pfizer may also provide access to the related individual de-identified participant data. For more information, go to https://www.pfizer.com/science/clinical-trials/trial-data-and-results.

## Abbreviations

CGRP: Calcitonin gene-related peptide
ECG: Electrocardiogram
MBS: Most bothersome symptom
MOH: Medication overuse headache
NSAID: Non-steroidal anti-inflammatory drug
ODT: Orally disintegrating tablet
TEAE: Treatment-emergent adverse event
