# Supplementary material for: Rimegepant orally disintegrating tablet 75 mg for acute treatment of migraine in adults from China: a subgroup analysis of a double-blind, randomized, placebo-controlled, phase 3 clinical trial
Source: J Headache Pain. 2024 Apr 16;25(1):57. doi: 10.1186/s10194-024-01731-4 (PMC11020209; doi:10.1186/s10194-024-01731-4)
Supplement: Supplementary file 1 — Additional file 1: Supplementary Table 1. [file 10194_2024_1731_MOESM1_ESM.docx]

| **Supplementary Table 1. Time-course of the proportion of participants with pain freedom, MBS freedom, pain relief, and normal function from 15 minutes to 48 hours post-dose.^a^** | | | | |
| --- | --- | --- | --- | --- |
| **Endpoint** | **Rimegepant 75 mg**  ***n* = 537** | **Placebo**  ***n* = 537** | **Risk Difference^b^**  **(95% CI)** | ***p* value^c^** |
| **Pain Freedom** |  |  |  |  |
| 15 minutes post-dose | 4 (0.7%) | 9 (1.7%) | -0.9 (-2.2, 0.4) | 0.1683 |
| 30 minutes post-dose | 5 (0.9%) | 6 (1.1%) | -0.2 (-1.4, 1.0) | 0.7739 |
| 45 minutes post-dose | 17 (3.2%) | 11 (2.0%) | 1.1 (-0.8, 3.0) | 0.2708 |
| 60 minutes post-dose | 32 (6.0%) | 24 (4.5%) | 1.5 (-1.1, 4.2) | 0.2567 |
| 90 minutes post-dose | 61 (11.4%) | 39 (7.3%) | 4.1 (0.7, 7.6) | 0.0204 |
| 2 hours post-dose | 98 (18.2%) | 57 (10.6%) | 7.6 (3.5, 11.8) | 0.0004 |
| 3 hours post-dose | 149 (27.7%) | 77 (14.3%) | 13.3 (8.5, 18.2) | < 0.0001 |
| 4 hours post-dose | 199 (37.1%) | 109 (20.3%) | 16.7 (11.4, 22.0) | < 0.0001 |
| 6 hours post-dose | 257 (47.9%) | 170 (31.7%) | 16.2 (10.4, 22.0) | < 0.0001 |
| 8 hours post-dose | 331 (61.6%) | 235 (43.8%) | 18.0 (12.1, 23.8) | < 0.0001 |
| 24 hours post-dose | 408 (76.0%) | 360 (67.0%) | 9.2 (3.8, 14.6) | 0.0008 |
| 48 hours post-dose | 445 (82.9%) | 394 (73.4%) | 9.6 (4.7, 14.5) | 0.0001 |
| **MBS Freedom** |  |  |  |  |
| 15 minutes post-dose | 35 (6.5%) | 36 (6.7%) | -0.1 (-3.1, 2.9) | 0.9349 |
| 30 minutes post-dose | 62 (11.5%) | 57 (10.6%) | 0.9 (-2.9, 4.6) | 0.6419 |
| 45 minutes post-dose | 102 (19.0%) | 83 (15.5%) | 3.5 (-1.0, 8.0) | 0.1306 |
| 60 minutes post-dose | 145 (27.0%) | 112 (20.9%) | 6.2 (1.1, 11.3) | 0.0168 |
| 90 minutes post-dose | 202 (37.6%) | 148 (27.6%) | 10.0 (4.4, 15.6) | 0.0005 |
| 2 hours post-dose | 258 (48.0%) | 171 (31.8%) | 16.2 (10.4, 22.0) | < 0.0001 |
| 3 hours post-dose | 296 (55.1%) | 213 (39.7%) | 15.6 (9.7, 21.5) | < 0.0001 |
| 4 hours post-dose | 328 (61.1%) | 244 (45.4%) | 15.7 (9.8, 21.6) | < 0.0001 |
| 6 hours post-dose | 370 (68.9%) | 306 (57.0%) | 12.0 (6.3, 17.8) | < 0.0001 |
| 8 hours post-dose | 418 (77.8%) | 340 (63.3%) | 14.7 (9.3, 20.1) | < 0.0001 |
| 24 hours post-dose | 449 (83.6%) | 404 (75.2%) | 8.6 (3.8, 13.4) | 0.0005 |
| 48 hours post-dose | 453 (84.4%) | 406 (75.6%) | 8.9 (4.2, 13.7) | 0.0003 |
| **Pain Relief** |  |  |  |  |
| 15 minutes post-dose | 44 (8.2%) | 56 (10.4%) | -2.2 (-5.7, 1.3) | 0.2108 |
| 30 minutes post-dose | 112 (20.9%) | 106 (19.7%) | 1.1 (-3.7, 5.9) | 0.6566 |
| 45 minutes post-dose | 184 (34.3%) | 139 (25.9%) | 8.4 (2.9, 13.9) | 0.0028 |
| 60 minutes post-dose | 239 (44.5%) | 167 (31.1%) | 13.6 (7.8, 19.3) | < 0.0001 |
| 90 minutes post-dose | 300 (55.9%) | 233 (43.4%) | 12.5 (6.6, 18.5) | < 0.0001 |
| 2 hours post-dose | 351 (65.4%) | 256 (47.7%) | 17.8 (12.0, 23.7) | < 0.0001 |
| 3 hours post-dose | 402 (74.9%) | 305 (56.8%) | 18.1 (12.5, 23.6) | < 0.0001 |
| 4 hours post-dose | 422 (78.6%) | 336 (62.6%) | 16.0 (10.6, 21.4) | < 0.0001 |
| 6 hours post-dose | 451 (84.0%) | 390 (72.6%) | 11.3 (6.4, 16.2) | < 0.0001 |
| 8 hours post-dose | 474 (88.3%) | 410 (76.4%) | 12.1 (7.6, 16.6) | < 0.0001 |
| 24 hours post-dose | 491 (91.4%) | 440 (81.9%) | 9.7 (5.7, 13.7) | < 0.0001 |
| 48 hours post-dose | 485 (90.3%) | 435 (81.0%) | 9.4 (5.3, 13.6) | < 0.0001 |
| **Normal Function^d^** |  |  |  |  |
| 15 minutes post-dose | 8 (1.8%) | 11 (2.4%) | -0.6 (-2.5, 1.2) | 0.5116 |
| 30 minutes post-dose | 37 (8.1%) | 28 (6.1%) | 2.1 (-1.2, 5.4) | 0.2181 |
| 45 minutes post-dose | 64 (14.0%) | 54 (11.7%) | 2.2 (-2.1, 6.6) | 0.3145 |
| 60 minutes post-dose | 94 (20.6%) | 71 (15.4%) | 5.2 (0.2, 10.2) | 0.0402 |
| 90 minutes post-dose | 124 (27.1%) | 94 (20.3%) | 6.8 (1.3, 12.3) | 0.0160 |
| 2 hours post-dose | 176 (38.5%) | 110 (23.8%) | 14.7 (8.8, 20.6) | < 0.0001 |
| 3 hours post-dose | 210 (46.0%) | 142 (30.7%) | 15.2 (9.0, 21.5) | < 0.0001 |
| 4 hours post-dose | 241 (52.7%) | 181 (39.2%) | 13.6 (7.2, 20.0) | < 0.0001 |
| 6 hours post-dose | 281 (61.5%) | 241 (52.2%) | 9.3 (3.0, 15.7) | 0.0043 |
| 8 hours post-dose | 341 (74.6%) | 283 (61.3%) | 13.5 (7.6, 19.5) | < 0.0001 |
| 24 hours post-dose | 382 (83.6%) | 363 (78.6%) | 5.1 (0.1, 10.2) | 0.0469 |
| 48 hours post-dose | 392 (85.8%) | 367 (79.4%) | 6.5 (1.6, 11.3) | 0.0097 |
| ^a^ Includes all randomized Chinese participants who took study treatment, had a migraine of moderate or severe intensity at the time of treatment, and provided at least one post-treatment efficacy data point. See methods section for details on handling of missing data and definition of failures.  ^b^ Rimegepant vs. placebo, calculated from Mantel–Haenszel test stratified by preventive migraine medication use.  ^c^ Rimegepant vs. placebo, calculated from Cochran-Mantel-Haenszel test stratified by preventive migraine medication use. All *p* values are nominal.  ^d^ Among participants with functional disability at time of dosing (rimegepant, *n* = 457; placebo, *n* = 462).  MBS, most bothersome symptom. | | | | |
