# Supplementary material for: Rimegepant orally disintegrating tablet 75 mg for acute treatment of migraine in adults from China: a subgroup analysis of a double-blind, randomized, placebo-controlled, phase 3 clinical trial
Source: J Headache Pain. 2024 Apr 16;25(1):57. doi: 10.1186/s10194-024-01731-4 (PMC11020209; doi:10.1186/s10194-024-01731-4)
Supplement: Supplementary file 2 — Additional file 2. English plain langauge summary. [file 10194_2024_1731_MOESM2_ESM.pdf]

Plain Language Summary

A study of the benefits and safety of rimegepant for treating a migraine attack in Chinese adults

Please note that this summary only contains information from the full scientific article:

View Scientific Article

Acetaminophen
<uh-see-tuh-MIH-nuh-fen>

Antiemetics
<AN-tee-uh-MEH-tiks>

Anti-inflammatory
<AN-tee-in-FLA-muh-TOR-ee>

Aura
<AW-ruh>

Calcitonin
<KAL-sih-TOH-nin >

Ergot alkaloids
<UR-guht AL-kuh-loydz>

Hemiplegic
<heh-meh-PLEE-jik>

Migraine
<MAI-grayn>

Nausea
<NAW-zee-uh>

Phonophobia
<FOW-nuh-FOH-bee-uh>

Photophobia
<FOW-toh-FOW-bee-uh>

Rimegepant
<rhe-MEG-je-pant >

Additional information

More information can be found in the scientific article of this study, which you can access here:

View Scientific Article

For more information on clinical studies in general, please visit:

https://www.clinicaltrials.gov/ct2/about-studies/learn

https://www.cancerresearchuk.org/about-cancer/find-a-clinical-trial/what-clinical-trials-are

Date of summary: December 2023
Study number: NCT04574362
Study start date: October 2020
Study end date: December 2021
The full title of this article: Rimegepant orally disintegrating tablet 75 mg for acute treatment of migraine in adults from China: A subgroup analysis of a double-blind, randomized, placebo-controlled, phase 3 clinical trial.

Key takeaways

- Researchers looked at the use of rimegepant for the treatment of a migraine attack in Chinese adults. Migraine is a condition with recurring headaches and other symptoms that affect a person's ability to perform normal daily activities.
- In this study, more people taking rimegepant were free of pain and their other most bothersome migraine symptom after 2 hours than people taking a placebo. Placebo is a harmless treatment that has no medical effect.
- People taking rimegepant had similar side effects as people taking a placebo.
- These results show that rimegepant may be a useful new medicine to treat migraine attacks in Chinese adults.

The purpose of this plain language summary is to help you to understand the findings from recent research.

- Rimegepant is used to treat migraine. Approval varies by country; please check with your local provider for more details.
- The results of this study may differ from those of other studies. Health professionals should make treatment decisions based on all available evidence, not on the results of a single study.

More information can be found in the scientific article of this study, which you can access here: View Scientific Article

What did this study look at?

Migraine

- Migraine is a condition that affects the nervous system.
  - The nervous system is made up of the brain, spinal cord, and a network of nerves that send signals back and forth between the brain and the body.
- The main symptom of migraine is usually pain on one side of the head.
  - Migraine pain is usually moderate or severe and happens repeatedly during a person's lifetime.
- Other common symptoms can include:
  - Feeling sick to your stomach (this is called "nausea").
  - Being sensitive to light (this is called "photophobia").
  - Being sensitive to noise (this is called "phonophobia").
- A migraine attack can last from a few hours to a few days.
- Migraine affects about 152 million people in China.
- There is a need for new migraine treatments in China because current treatments (such as triptans, non-steroidal anti-inflammatory drugs, and ergot alkaloids) may:
  - Be unsafe for use in people with heart disease.
  - Cause headache if used too much.
  - Not be effective for migraine attacks with severe pain.

What is rimegepant?

- Rimegepant is a medicine used to treat migraine.
  - It is a tablet that is taken by mouth and dissolves in a person's mouth.
  - It can be taken to stop a migraine that has already started.
    - This is called "acute treatment".
  - It can also be taken every other day to help prevent migraine attacks.
- Rimegepant binds to calcitonin gene-related peptide (CGRP) receptors.
  - CGRP is a small protein produced within the body.
  - Receptors are proteins on the surface of a cell, or within a cell, that respond to specific chemicals, proteins, or other substances.
  - A key step in the development of migraine is CGRP sticking to receptors in and around the brain.
    - When CGRP sticks to the receptors, it triggers signals within the brain that lead to symptoms of migraine.
  - Rimegepant blocks CGRP from sticking to receptors and prevents the signals that cause migraine symptoms.

Acetaminophen
<uh-see-tuh-MIH-nuh-fen>

Antiemetics
<AN-tee-uh-MEH-tiks>

Anti-inflammatory
<AN-tee-in-FLA-muh-TOR-ee>

Aura
<AW-ruh>

Calcitonin
<KAL-sih-TOH-nin >

Ergot alkaloids
<UR-guht AL-kuh-loydz>

Hemiplegic
<heh-meh-PLEE-jik>

Migraine
<MAI-grayn>

Nausea
<NAW-zee-uh>

Phonophobia
<FOW-nuh-FOH-bee-uh>

Photophobia
<FOW-toh-FOW-bee-uh>

Rimegepant
<rhe-MEG-je-pant >

Additional information

More information can be found in the scientific article of this study, which you can access here:

View Scientific Article

For more information on clinical studies in general, please visit:

https://www.clinicaltrials.gov/ct2/about-studies/learn

https://www.cancerresearchuk.org/about-cancer/find-a-clinical-trial/what-clinical-trials-are

Acetaminophen
<uh-see-tuh-MIH-nuh-fen>

Antiemetics
<AN-tee-uh-MEH-tiks>

Anti-inflammatory
<AN-tee-in-FLA-muh-TOR-ee>

Aura
<AW-ruh>

Calcitonin
<KAL-sih-TOH-nin >

Ergot alkaloids
<UR-guht AL-kuh-loydz>

Hemiplegic
<heh-meh-PLEE-jik>

Migraine
<MAI-grayn>

Nausea
<NAW-zee-uh>

Phonophobia
<FOW-nuh-FOH-bee-uh>

Photophobia
<FOW-toh-FOW-bee-uh>

Rimegepant
<rhe-MEG-je-pant >

Additional information

More information can be found in the scientific article of this study, which you can access here:

View Scientific Article

For more information on clinical studies in general, please visit:

https://www.clinicaltrials.gov/ct2/about-studies/learn

https://www.cancerresearchuk.org/about-cancer/find-a-clinical-trial/what-clinical-trials-are

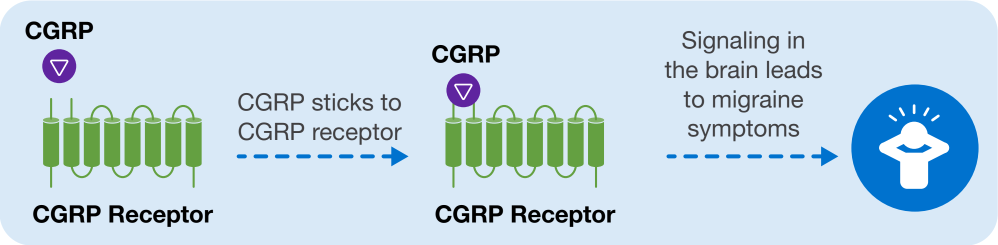

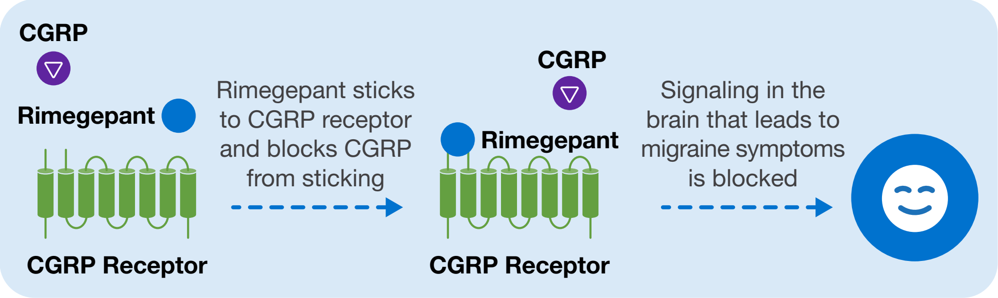

What was this study about?

- This study looked at the benefits and side effects of a single dose of rimegepant taken to treat a single migraine attack with moderate or severe pain in adults living in China.
- The main aims of the study were to see how many people had:
  - No pain 2 hours after taking rimegepant or a placebo.
    - A placebo does not contain any active ingredients.
    - The placebo and study medicine look alike.
    - A placebo is taken in the same way as the study medicine.
  - Freedom from their most bothersome other migraine symptom 2 hours after taking rimegepant or a placebo.
    - People had to choose from feeling sick to your stomach (this is called “nausea”), being sensitive to light (this is called “photophobia”), or being sensitive to noise (this is called “phonophobia”) as their most bothersome other migraine symptom.

Acetaminophen
<uh-see-tuh-MIH-nuh-fen>

Antiemetics
<AN-tee-uh-MEH-tiks>

Anti-inflammatory
<AN-tee-in-FLA-muh-TOR-ee>

Aura
<AW-ruh>

Calcitonin
<KAL-sih-TOH-nin >

Ergot alkaloids
<UR-guht AL-kuh-loydz>

Hemiplegic
<heh-meh-PLEE-jik>

Migraine
<MAI-grayn>

Nausea
<NAW-zee-uh>

Phonophobia
<FOW-nuh-FOH-bee-uh>

Photophobia
<FOW-toh-FOW-bee-uh>

Rimegepant
<rhe-MEG-je-pant >

Additional information

More information can be found in the scientific article of this study, which you can access here:

View Scientific Article

For more information on clinical studies in general, please visit:

https://www.clinicaltrials.gov/ct2/about-studies/learn

https://www.cancerresearchuk.org/about-cancer/find-a-clinical-trial/what-clinical-trials-are

How was this study conducted?

- People waited until a migraine attack with moderate or severe pain had started.
- People then answered questions about their migraine just before they took treatment. They were asked to:
  - Rate their level of pain.
  - Identify their other most bothersome migraine symptom.
  - Rate their ability to perform daily activities.
- People had to choose one of the following as their other most bothersome migraine symptom:
  - Feeling sick to their stomach (nausea).
  - Being sensitive to light (photophobia).
  - Being sensitive to sound (phonophobia).
- Immediately after answering these questions, people took 1 tablet of rimegepant or a placebo to treat their migraine attack.
- People continued to rate their level of pain, the presence of their other most bothersome migraine symptom, and ability to perform daily activities from 15 minutes to 48 hours after taking rimegepant or a placebo.

1 People have a migraine attack with moderate or severe pain

2 People rate their migraine symptoms just before taking treatment

What is their pain intensity?

0	1	2	3
None	Mild	Moderate	Severe

What is their other most bothersome migraine symptom?

Nausea | Photophobia | Phonophobia

What is their ability to function?

0	1	2	3
Normal function	Mildly impaired	Severely impaired	Requires bedrest

3 People take **rimegepant** or **placebo** to treat their migraine attack

4 People rate migraine symptoms at 15 minutes, 30 minutes, 45 minutes, 1 hour, 1.5 hours, 2 hours, 3 hours, 4 hours, 6 hours, 8 hours, 24 hours (1 day), and 48 hours (2 days) after treatment

What is their pain intensity?

0	1	2	3
None	Mild	Moderate	Severe

Is other most bothersome migraine symptom present?

0	1
Absent	Present

What is their ability to function?

0	1	2	3
Normal function	Mildly impaired	Severely impaired	Requires bedrest

- If people did not feel relief from their migraine 2 hours after taking rimegepant or a placebo they could take other medicines (this is called “rescue medication”), including:
  - Medicines to help stop pain (aspirin, ibuprofen, acetaminophen, non-steroidal anti-inflammatory drugs).
  - Medicines to stop stomach sickness and the urge to vomit (these are called “antiemetics”).
  - Or a medicine that relaxes muscles (known as baclofen).

Acetaminophen
<uh-see-tuh-MIH-nuh-fen>

Antiemetics
<AN-tee-uh-MEH-tiks>

Anti-inflammatory
<AN-tee-in-FLA-muh-TOR-ee>

Aura
<AW-ruh>

Calcitonin
<KAL-sih-TOH-nin >

Ergot alkaloids
<UR-guht AL-kuh-loydz>

Hemiplegic
<heh-meh-PLEE-jik>

Migraine
<MAI-grayn>

Nausea
<NAW-zee-uh>

Phonophobia
<FOW-nuh-FOH-bee-uh>

Photophobia
<FOW-toh-FOW-bee-uh>

Rimegepant
<rhe-MEG-je-pant >

Additional information

More information can be found in the scientific article of this study, which you can access here:

View Scientific Article

For more information on clinical studies in general, please visit:

https://www.clinicaltrials.gov/ct2/about-studies/learn

https://www.cancerresearchuk.org/about-cancer/find-a-clinical-trial/what-clinical-trials-are

Who took part in this study?

- People who took part in the study:
  - Lived in China.
  - Men and women at least 18 years old.
  - Had a history of migraines for at least 1 year.
  - Had 2 to 8 migraines of moderate or severe pain intensity each month at least 3 months before the study started.
  - Had migraines lasting from 4 hours to 72 hours (3 days) if left untreated.
- People could not take part in the study if they had certain medical conditions or used medicines that would make it difficult or unsafe for them to take part. Also, people could not take part if they:
  - Had migraine with brainstem aura.
    - Aura is a group of temporary nervous system symptoms that usually occur before headache and other symptoms of migraine.
    - Brainstem aura means the symptoms come from the base of the brain or from both sides of the brain at the same time. Examples include difficulty speaking, double vision, ringing in the ears, feeling unsteady, and feeling like things around you are spinning.
  - Had hemiplegic migraine.
    - This is a type of migraine that causes weakness on one side of the body.
  - Participated in a study of other medicines that bind CGRP receptors.
  - Took CGRP antibodies within the last 6 months.
    - CGRP antibodies are a type of medicine that doctors sometimes give patients to prevent migraine.

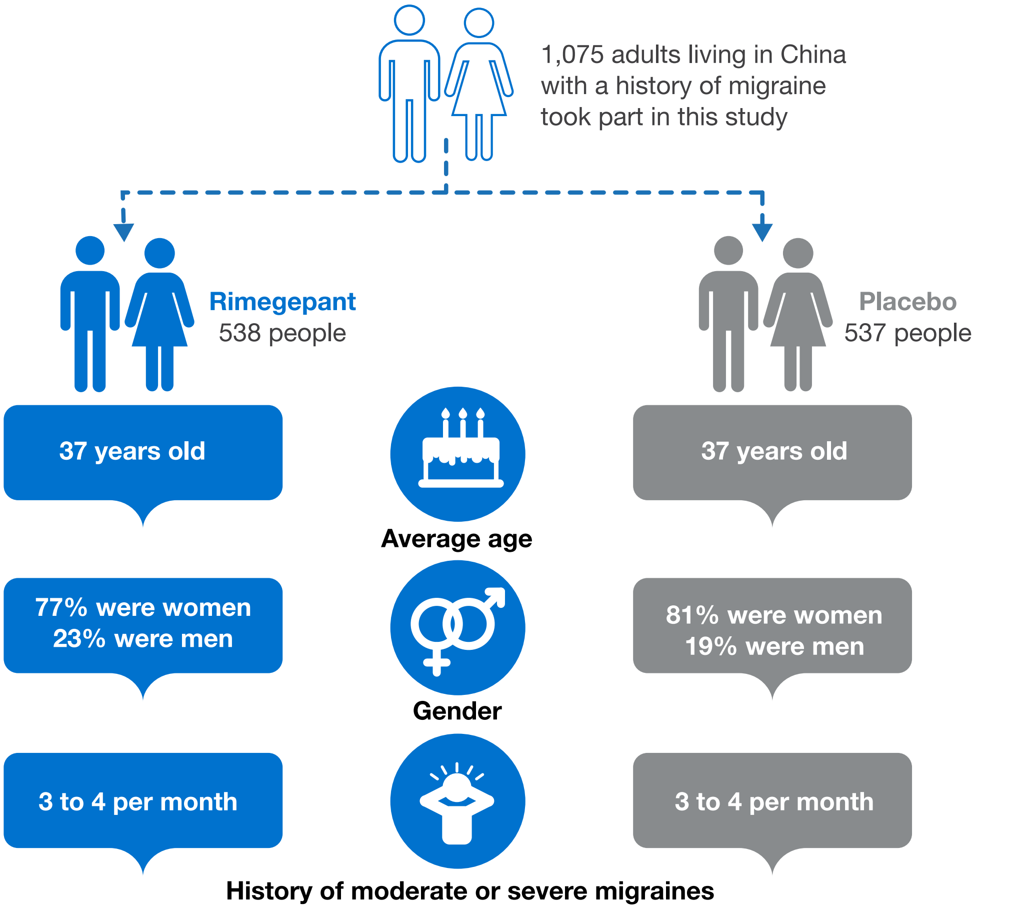

Age, gender, and the average number of migraines per month before entering the study were similar in people who took **rimegepant** and people who took **placebo**

Acetaminophen
<uh-see-tuh-MIH-nuh-fen>

Antiemetics
<AN-tee-uh-MEH-tiks>

Anti-inflammatory
<AN-tee-in-FLA-muh-TOR-ee>

Aura
<AW-ruh>

Calcitonin
<KAL-sih-TOH-nin >

Ergot alkaloids
<UR-guht AL-kuh-loydz>

Hemiplegic
<heh-meh-PLEE-jik>

Migraine
<MAI-grayn>

Nausea
<NAW-zee-uh>

Phonophobia
<FOW-nuh-FOH-bee-uh>

Photophobia
<FOW-toh-FOW-bee-uh>

Rimegepant
<rhe-MEG-je-pant >

Additional information

More information can be found in the scientific article of this study, which you can access here:

View Scientific Article

For more information on clinical studies in general, please visit:

https://www.clinicaltrials.gov/ct2/about-studies/learn

https://www.cancerresearchuk.org/about-cancer/find-a-clinical-trial/what-clinical-trials-are

Results of the study

How well did the treatments (rimegepant or a placebo) work?

- Two hours after taking rimegepant or a placebo, more people taking rimegepant had:
  - **Pain freedom** (this means they had no pain).
  - **Freedom from their other most bothersome migraine symptom** (this means the symptom, other than pain, that bothered them the most just before taking treatment was gone).
  - **Pain relief** (this means they had no pain or had only mild pain).
  - **Normal function** (this means they could do daily activities as they normally would).

People took 1 tablet of rimegepant or a placebo when they had a migraine attack with moderate or severe pain.

People rated their level of pain, presence of their other most bothersome symptom, and their ability to function just before taking treatment and 2 hours after taking treatment.

	Proportion of people with **pain freedom** (no pain)	Proportion of people with **freedom from the most bothersome symptom** (symptom is gone)	Proportion of people with **pain relief** (no or mild pain)	Proportion of people with **normal function** (can do daily activities as normal)
people who took **rimegepant**	18% 98 of 537	48% 258 of 537	65% 351 of 537	39% 176 of 457
people who took **placebo**	11% 57 of 537	32% 171 of 537	48% 256 of 537	24% 110 of 462

Acetaminophen
<uh-see-tuh-MIH-nuh-fen>

Antiemetics
<AN-tee-uh-MEH-tiks>

Anti-inflammatory
<AN-tee-in-FLA-muh-TOR-ee>

Aura
<AW-ruh>

Calcitonin
<KAL-sih-TOH-nin >

Ergot alkaloids
<UR-guht AL-kuh-loydz>

Hemiplegic
<heh-meh-PLEE-jik>

Migraine
<MAI-grayn>

Nausea
<NAW-zee-uh>

Phonophobia
<FOW-nuh-FOH-bee-uh>

Photophobia
<FOW-toh-FOW-bee-uh>

Rimegepant
<rhe-MEG-je-pant >

- More people who took rimegepant had **sustained pain freedom from 2 to 24 hours** than people who took a placebo.
  - This means they had no pain at any time from 2 hours after taking rimegepant or a placebo to 1 day after taking rimegepant or a placebo.
- More people who took rimegepant had **sustained pain freedom from 2 to 48 hours** than people who took a placebo.
  - This means they had no pain at any time from 2 hours after taking rimegepant or a placebo to 2 days after taking rimegepant or a placebo.

People took 1 tablet of rimegepant or a placebo when they had a migraine attack with moderate or severe pain.

People rated their level of pain just before taking treatment and at 2 hours, 3 hours, 4 hours, 6 hours, 8 hours, 24 hours (1 day), and 48 hours (2 days) after taking treatment.

Proportion of people with **sustained pain freedom from 2 to 24 hours** (no pain at any time from 2 hours after taking rimegepant or a placebo to 1 day after taking rimegepant or a placebo)

15%
82 of 537
people who took **rimegepant**

8%
43 of 537
people who took **placebo**

Proportion of people with **sustained pain freedom from 2 to 48 hours** (no pain at any time from 2 hours after taking rimegepant or a placebo to 2 days after taking rimegepant or a placebo)

15%
78 of 537
people who took **rimegepant**

7%
39 of 537
people who took **placebo**

- Fewer people who took rimegepant needed to **use other medicines for their migraine (rescue medication) during the 24 hours (1 day)** after taking rimegepant than people who took a placebo.

People took 1 tablet of rimegepant or a placebo when they had a migraine attack with moderate or severe pain.

People kept track of other medicines for migraine that they used during the 24 hours (1 day) after taking treatment.

Proportion of people who **used other medicines for migraine (rescue medication) during the 24 hours (1 day)** after taking rimegepant or a placebo

5%
28 of 537
people who took **rimegepant**

14%
75 of 537
people who took **placebo**

Additional information

More information can be found in the scientific article of this study, which you can access here:

View Scientific Article

For more information on clinical studies in general, please visit:

https://www.clinicaltrials.gov/ct2/about-studies/learn

https://www.cancerresearchuk.org/about-cancer/find-a-clinical-trial/what-clinical-trials-are

Acetaminophen
<uh-see-tuh-MIH-nuh-fen>

Antiemetics
<AN-tee-uh-MEH-tiks>

Anti-inflammatory
<AN-tee-in-FLA-muh-TOR-ee>

Aura
<AW-ruh>

Calcitonin
<KAL-sih-TOH-nin >

Ergot alkaloids
<UR-guht AL-kuh-loydz>

Hemiplegic
<heh-meh-PLEE-jik>

Migraine
<MAI-grayn>

Nausea
<NAW-zee-uh>

Phonophobia
<FOW-nuh-FOH-bee-uh>

Photophobia
<FOW-toh-FOW-bee-uh>

Rimegepant
<rhe-MEG-je-pant >

How safe were the treatments (rimegepant or a placebo)?

- The number of people with **side effects** was similar for people who took rimegepant and people who took a placebo.
  - Side effects are symptoms or medical conditions that start or worsen after someone starts a treatment. Side effects may or may not be related to the treatment.
  - The most common side effect among people taking rimegepant or placebo during the study was feeling sick to their stomach (also called “nausea”).

People took 1 tablet of rimegepant or a placebo when they had a migraine attack with moderate or severe pain.

People kept track of side effects they had after taking treatment.

Proportion of people who **had any side effect** after taking rimegepant or a placebo

15% 82 of 538 people who took **rimegepant**

16% 88 of 537 people who took **placebo**

Proportion of people who **felt sick to their stomach (nausea)** after taking rimegepant or a placebo

1% 6 of 538 people who took **rimegepant**

3% 14 of 537 people who took **placebo**

- Less than 1% of people who took rimegepant (1 of 538 people) or a placebo (2 of 537 people) had a side effect that researchers thought was serious.
  - A side effect is typically considered serious when it is life-threatening, needs hospital care, causes lasting problems, or needs medical or surgical treatment.

- More results from this study can be found here: **View Scientific Article**

Additional information

More information can be found in the scientific article of this study, which you can access here:

View Scientific Article

For more information on clinical studies in general, please visit:

https://www.clinicaltrials.gov/ct2/about-studies/learn

https://www.cancerresearchuk.org/about-cancer/find-a-clinical-trial/what-clinical-trials-are

What were the main conclusions reported by the researchers?

- In this study, more people who took rimegepant were free of pain and their other most bothersome migraine symptom after 2 hours than people who took a placebo.
- People who took rimegepant and people who took a placebo had similar side effects.
- These results show that rimegepant may be a useful new medicine to treat migraine attacks in Chinese adults.
- However, the study only looked at effects of a single dose of rimegepant.
  - Conclusions on repeated long-term use of rimegepant cannot be made from this study.

Are there plans for additional studies?

Additional studies of rimegepant are planned. These studies will help researchers further understand the long-term benefits and safety of rimegepant for treatment of migraine in Chinese patients.

Where can I find more information?

This study looked at the use of rimegepant to treat migraine in people living in China or South Korea. The article summarized here includes results for people living in China. Results for all people in the study (China and South Korea) have been published. You can access the previous article here:
View Previous Scientific Article.

For more information on this study, please visit:

https://www.clinicaltrials.gov/study/NCT04574362

For more information on clinical studies in general, please visit:

https://www.clinicaltrials.gov/ct2/about-studies/learn

Who sponsored this study?

This study was sponsored by BioShin, a wholly owned subsidiary of Biohaven Pharmaceuticals. Biohaven Pharmaceuticals was acquired by Pfizer in October 2022.

Pfizer Inc.

235 East 42nd Street NY, NY 10017

Phone (United States): +1 212-733-232

Pfizer thanks everyone who took part in this study.

Acknowledgments

Writing support for this summary was provided by Matt Soulsby, PhD, CMPP, at Envision Pharma Group, and was funded by Pfizer. Authors of the full article were involved in preparing this summary.

Acetaminophen
<uh-see-tuh-MIH-nuh-fen>

Antiemetics
<AN-tee-uh-MEH-tiks>

Anti-inflammatory
<AN-tee-in-FLA-muh-TOR-ee>

Aura
<AW-ruh>

Calcitonin
<KAL-sih-TOH-nin >

Ergot alkaloids
<UR-guht AL-kuh-loydz>

Hemiplegic
<heh-meh-PLEE-jik>

Migraine
<MAI-grayn>

Nausea
<NAW-zee-uh>

Phonophobia
<FOW-nuh-FOH-bee-uh>

Photophobia
<FOW-toh-FOW-bee-uh>

Rimegepant
<rhe-MEG-je-pant >

Additional information

More information can be found in the scientific article of this study, which you can access here:

View Scientific Article

For more information on clinical studies in general, please visit:

https://www.clinicaltrials.gov/ct2/about-studies/learn

https://www.cancerresearchuk.org/about-cancer/find-a-clinical-trial/what-clinical-trials-are
