# Supplementary material for: Rimegepant orally disintegrating tablet 75 mg for acute treatment of migraine in adults from China: a subgroup analysis of a double-blind, randomized, placebo-controlled, phase 3 clinical trial
Source: J Headache Pain. 2024 Apr 16;25(1):57. doi: 10.1186/s10194-024-01731-4 (PMC11020209; doi:10.1186/s10194-024-01731-4)
Supplement: Supplementary file 3 — Additional file 3. Chinese plain langauge summary. [file 10194_2024_1731_MOESM3_ESM.pdf]

通俗语言摘要

# 瑞美吉泮治疗中国成人偏头痛发作的益处和安全性研究

请注意，本摘要仅包含科研文章全文中的信息：

查看科研文章

**摘要日期:** 2023 年 12 月

**研究编号:** NCT04574362

**研究开始日期:** 2020 年 10 月

**研究结束日期:** 2021 年 12 月

**本文章的完整标题:** 瑞美吉泮口腔崩解片（75 毫克）急性治疗中国成人偏头痛： 一项双盲、随机、安慰剂对照3期临床试验的亚组分析。

## 要点

- 研究人员调查了中国成年人使用瑞美吉泮治疗偏头痛发作的情况。偏头痛是一种反复发作的头痛，常伴随其他症状，会影响患者的日常活动能力。
- 在这项研究中，与服用安慰剂的人相比，更多的人在服用瑞美吉泮两小时后摆脱了疼痛和其他最令人困扰的偏头痛症状。安慰剂是一种无害的物质，没有任何治疗效果。
- 服用瑞美吉泮的人与服用安慰剂的人出现的副作用相似。
- 这些结果表明，瑞美吉泮可能是治疗中国成年人偏头痛发作的有效新药。

**本摘要采用通俗语言，旨在帮助您了解最新研究成果。**

- 瑞美吉泮用于治疗偏头痛。批准情况因国家而异，详情请咨询当地供应商。
- 本研究的结果可能与其他研究的结果不同。医疗专业人员应根据所有可用的证据来做出治疗决定，而不是仅通过单项研究的结果。

欲了解更多信息，请点击此处查阅此研究的科研文章：查看科研文章

对乙酰氨基酚（**Acetaminophen**）
<Duì Yǐ Xiān Ān Jī Fēn>

止吐药（**Antiemetics**）
<Zhǐ Tù Yào>

抗炎药（**Anti-inflammatory**）
<Kàng Yán Yào>

先兆
<Xiān Zhào>

降钙素（**Calcitonin**）
<Jiàng Gài Sù>

麦角生物碱（**Ergot alkaloids**）
<Mài Jiǎo Shēng Wù Jiǎn>

偏瘫（**Hemiplegic**）
<Piān Tān>

偏头痛（**Migraine**）
<Piān Tóu Tòng>

恶心（**Nausea**）
<Ě Xīn>

恐声症（**Phonophobia**）
<Kǒng Shēng Zhèng>

畏光（**Photophobia**）
<Wèi Guāng>

瑞美吉泮（**Rimegepant**）
<Ruì Měi Jí Pàn>

## 更多信息

**欲了解更多信息，请点击此处查阅此研究的科研文章：**

查看科研文章

**如需了解有关各方面临床研究的更多信息，请访问：**

https://www.clinicaltrials.gov/ct2/about-studies/learn

https://www.cancerresearchuk.org/about-cancer/find-a-clinical-trial/what-clinical-trials-are

# 本研究的内容是什么?

## 偏头痛 (Migraine)

- 偏头痛是一种影响神经系统的疾病
  - 神经系统由大脑、脊髓以及在大脑和身体之间来回传递信号的神经网络组成。
- 偏头痛的主要症状通常是头部一侧疼痛。
  - 偏头痛通常为中度或重度疼痛，在人的一生中会反复发作。
- 其他常见症状还包括：
  - 感到胃部不适（称为"恶心"）。
  - 对光线敏感（称为"畏光"）。
  - 对声音敏感（称为"畏声"）。
- 偏头痛发作可持续几个小时到几天不等。
- 中国约有 1.52 亿人患有偏头痛。
- 中国需要新的偏头痛治疗方法，因为目前的治疗方法（如三苯氧胺、非甾体抗炎药和麦角生物碱）具有局限性：
  - 在心脏病患者中使用不安全。
  - 过度使用还会引发头痛。
  - 对严重的偏头痛发作可能无效。

## 瑞美吉泮是什么?

- 瑞美吉泮是用于治疗偏头痛的药物。
  - 它是一种口服片剂，可在人的口腔中溶解。
  - 偏头痛已经开始发作时，可以服用它来止痛。
    - 这被称为"急性治疗"。
  - 它也可以隔天服用一次，来预防偏头痛发作。
- 瑞美吉泮可与降钙素基因相关肽（CGRP）受体结合。
  - CGRP 是人体内产生的一种小分子蛋白质。
  - 受体是细胞表面或细胞内的蛋白质，能对特定的化学物质、蛋白质或其他物质做出反应。
  - 偏头痛发病的一个关键步骤是 CGRP 结合在大脑内部和周围的 CGRP受体上。
    - 当CGRP结合在CGRP受体上时，就会触发大脑内的信号，从而导致偏头痛发作。
  - 瑞美吉泮可阻断CGRP结合到CGRP受体上，从而阻止引发偏头痛发作的信号。

对乙酰氨基酚（**Acetaminophen**）
<Duì Yǐ Xiān Ān Jī Fēn>

止吐药（**Antiemetics**）
<Zhǐ Tù Yào>

抗炎药（**Anti-inflammatory**）
<Kàng Yán Yào>

先兆
<Xiān Zhào>

降钙素（**Calcitonin**）
<Jiàng Gài Sù>

麦角生物碱（**Ergot alkaloids**）
<Mài Jiǎo Shēng Wù Jiǎn>

偏瘫（**Hemiplegic**）
<Piān Tān>

偏头痛（**Migraine**）
<Piān Tóu Tòng>

恶心（**Nausea**）
<Ě Xīn>

恐声症（**Phonophobia**）
<Kǒng Shēng Zhèng>

畏光（**Photophobia**）
<Wèi Guāng>

瑞美吉泮（**Rimegepant**）
<Ruì Měi Jí Pàn>

## 更多信息

**欲了解更多信息，请点击此处查阅此研究的科研文章：**

**查看科研文章**

**如需了解有关各方面临床研究的更多信息，请访问：**

https://www.clinicaltrials.gov/ct2/about-studies/learn

https://www.cancerresearchuk.org/about-cancer/find-a-clinical-trial/what-clinical-trials-are

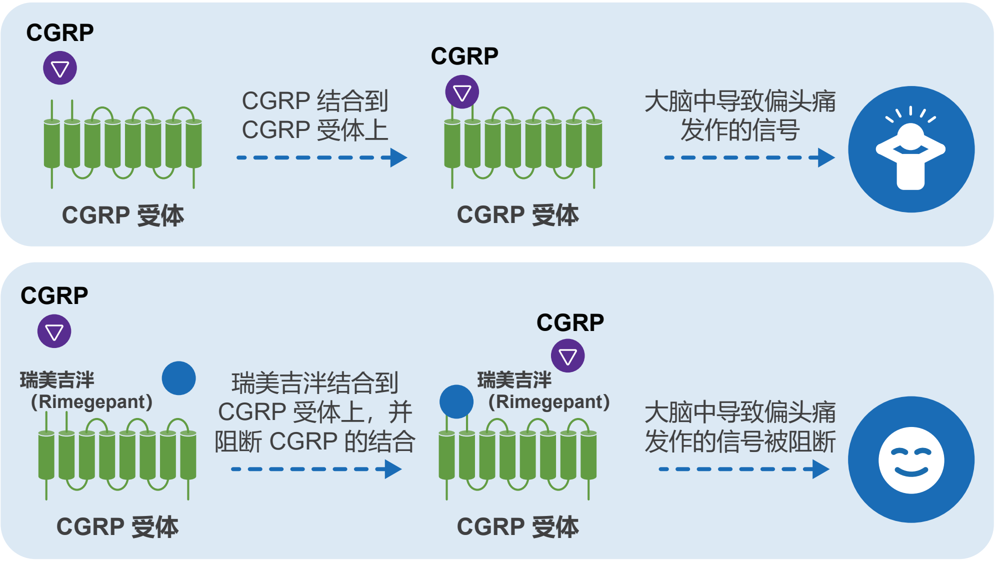


对乙酰氨基酚（**Acetaminophen**）
<Duì Yĭ Xiān Ān Jī Fēn>

止吐药（**Antiemetics**）
<Zhĭ Tù Yào>

抗炎药（**Anti-inflammatory**）
<Kàng Yán Yào>

先兆
<Xiān Zhào>

降钙素（**Calcitonin**）
<Jiàng Gài Sù>

麦角生物碱（**Ergot alkaloids**）
<Mài Jiăo Shēng Wù Jiăn>

偏瘫（**Hemiplegic**）
<Piān Tān>

偏头痛（**Migraine**）
<Piān Tóu Tòng>

恶心（**Nausea**）
<Ě Xīn>

恐声症（**Phonophobia**）
<Kŏng Shēng Zhèng>

畏光（**Photophobia**）
<Wèi Guāng>

瑞美吉泮（**Rimegepant**）
<Ruì Mĕi Jí Pàn>

## 这项研究与什么有关?

- 这项研究调查了中国成年人服用单剂量瑞美吉泮治疗中度或重度偏头痛发作的益处和副作用。
- 这项研究的主要目的是了解有多少人能够：
  - 在服用瑞美吉泮或安慰剂 2 小时后无疼痛。
    - 安慰剂不含任何活性成分。
    - 安慰剂和研究药物外观相似。
    - 安慰剂的服用方法与研究药物相同。
  - 在服用瑞美吉泮或安慰剂 2 小时后，不再出现最让他们困扰的其他偏头痛症状。
    - 受试者要选择以下一项作为他们感到最困扰的其他偏头痛症状，其中包括胃部不适（称为 “恶心” ）、对光敏感（称为 “畏光” ）或对噪音敏感（称为 “畏声” ）。

## 更多信息

**欲了解更多信息，请点击此处查阅此研究的科研文章：**

**查看科研文章**

**如需了解有关各方面临床研究的更多信息，请访问：**

**https://www.clinicaltrials.gov/ct2/about-studies/learn**

**https://www.cancerresearchuk.org/about-cancer/find-a-clinical-trial/what-clinical-trials-are**

对乙酰氨基酚（**Acetaminophen**）
<Duì Yǐ Xiān Ān Jī Fēn>

止吐药（**Antiemetics**）
<Zhǐ Tù Yào>

抗炎药（**Anti-inflammatory**）
<Kàng Yán Yào>

先兆
<Xiān Zhào>

降钙素（**Calcitonin**）
<Jiàng Gài Sù>

麦角生物碱（**Ergot alkaloids**）
<Mài Jiǎo Shēng Wù Jiǎn>

偏瘫（**Hemiplegic**）
<Piān Tān>

偏头痛（**Migraine**）
<Piān Tóu Tòng>

恶心（**Nausea**）
<Ě Xīn>

恐声症（**Phonophobia**）
<Kǒng Shēng Zhèng>

畏光（**Photophobia**）
<Wèi Guāng>

瑞美吉泮（**Rimegepant**）
<Ruì Měi Jí Pàn>

## 更多信息

**欲了解更多信息，请点击此处查阅此研究的科研文章：**

**查看科研文章**

**如需了解有关各方面临床研究的更多信息，请访问：**

**https://www.clinicaltrials.gov/ct2/about-studies/learn**

**https://www.cancerresearchuk.org/about-cancer/find-a-clinical-trial/what-clinical-trials-are**

# 这项研究是如何开展的？

- 受试者等待偏头痛发作并直至出现中度或重度疼痛。
- 在接受治疗前，受试者需回答有关偏头痛的问题。问题包括：
  - 他们的疼痛程度。
  - 描述最令他们困扰的其他偏头痛症状。
  - 评估他们进行日常活动的能力。
- 受试者需要选择下列症状中的一项作为最令他们困扰的其他偏头痛症状：
  - 感到胃部不适（恶心）。
  - 对光线敏感（畏光）。
  - 对声音敏感（畏声）。
- 回答完这些问题后，受试者立即服用一片瑞美吉泮或安慰剂来治疗他们的偏头痛发作。
- 受试者在服用瑞美吉泮或安慰剂后的 15 分钟至 48 小时，持续对自己的疼痛程度、其他最困扰的偏头痛症状以及日常活动能力进行评分。

**1** 患者偏头痛发作并伴有中度或重度疼痛

**他们感受到的疼痛程度如何？**

| 0 | 1 | 2 | 3 |
|---|---|---|---|
| 没有 | 轻度 | 中度 | 重度 |

**他们最困扰的其他偏头痛症状是什么？**

| 恶心 | 畏光 | 畏声 |
|---|---|---|
| (Nausea) | (Photophobia) | (Phonophobia) |

**2** 受试者在接受治疗前评估偏头痛症状

**他们开展日常活动的能力如何？**

| 0 | 1 | 2 | 3 |
|---|---|---|---|
| 功能正常 | 轻度受损 | 严重受损 | 需要卧床休息 |

**3** 受试者服用瑞美吉泮或安慰剂来治疗偏头痛发作

**他们感受到的疼痛程度如何？**

| 0 | 1 | 2 | 3 |
|---|---|---|---|
| 没有 | 轻度 | 中度 | 重度 |

**最困扰的其他偏头痛症状是否依然存在？**

| 0 | 1 |
|---|---|
| 不存在 | 存在 |

**4** 受试者在接受治疗后的 15 分钟、30 分钟、45 分钟、1 小时、1.5 小时、2 小时、3 小时、4 小时、6 小时、8 小时、24 小时（1 天）和 48 小时（2 天）时分别对偏头痛症状进行评分

**他们开展日常活动的能力如何？**

| 0 | 1 | 2 | 3 |
|---|---|---|---|
| 功能正常 | 轻度受损 | 严重受损 | 需要卧床休息 |

- 如果在服用瑞美吉泮或安慰剂 2 小时后，偏头痛仍未缓解，他们可以服用其他药物（称为“补救药物”），包括：
  - 止痛药（阿司匹林、布洛芬、对乙酰氨基酚、非甾体抗炎药）。
  - 用于阻止胃部不适和呕吐冲动的药物（称为 “止吐药”）。
  - 或者放松肌肉的药物（称为巴氯芬）。

# 谁参加了这项研究？

- 参加本次研究的人：
  – 居住在中国。
  – 男性和女性，年龄至少18岁。
  – 偏头痛病史至少1年。
  – 在研究开始前至少 3 个月，每月有 2 至 8 次中度或重度偏头痛发作。
  – 如果不进行治疗，偏头痛会持续 4 小时到 72 小时（3 天）。
- 如果患有某些疾病或服用某些药物导致患者参加本研究困难或存在安全隐患，那么这类患者将不能参加本研究。此外，下列情况的患者也无法参加本研究：
  – 患有脑干先兆偏头痛。
    - 先兆是一组短暂的神经系统症状，通常发生在头痛和偏头痛的其他症状之前。
    - 脑干先兆是指症状来自大脑底部或同时来自大脑两侧。例如，说话困难、重影、耳鸣、感觉不稳，以及感觉周围的事物在旋转。
  – 患有偏瘫性偏头痛。
    - 这是偏头痛的一种类型，会导致身体一侧无力。
  – 已经参与了其他与 CGRP 受体结合的药物的研究。
  – 在过去 6 个月内使用过 CGRP 抗体。
    - CGRP 抗体是一种药物，医生有时会给病人服用这种药物来预防偏头痛。

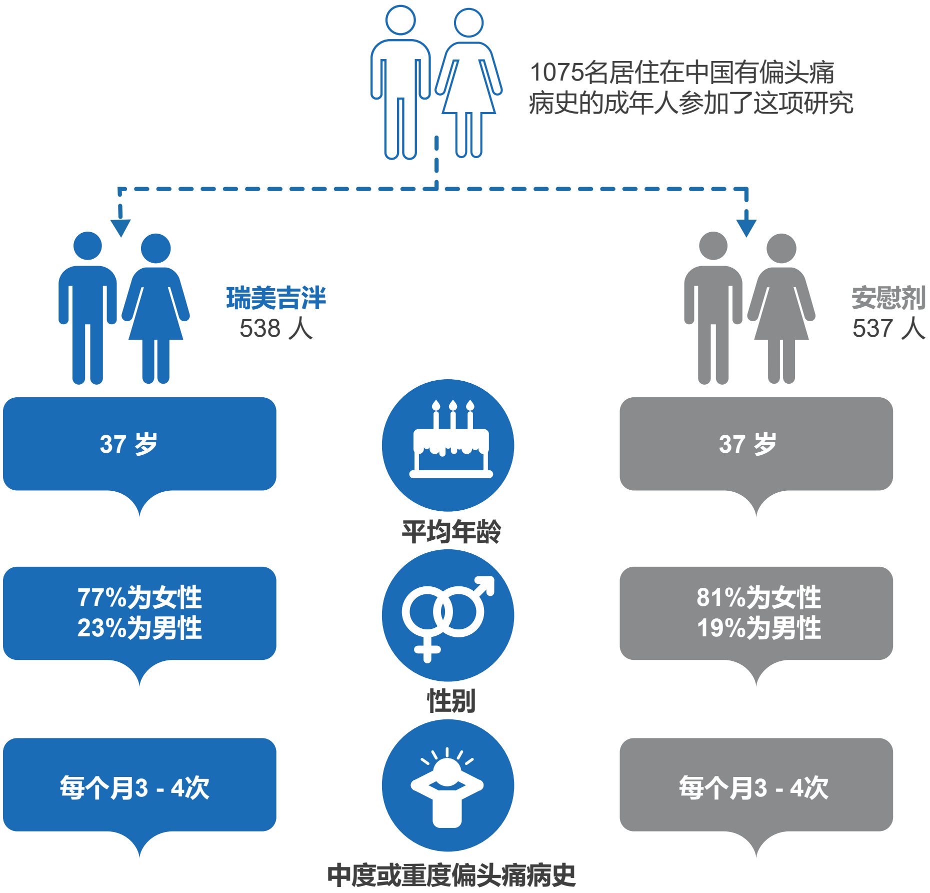


服用瑞美吉泮和服用安慰剂的人的年龄、性别和参加研究前每月偏头痛发作的平均次数相似

对乙酰氨基酚（**Acetaminophen**）
<Duì Yǐ Xiān Ān Jī Fēn>

止吐药（**Antiemetics**）
<Zhǐ Tù Yào>

抗炎药（**Anti-inflammatory**）
<Kàng Yán Yào>

先兆
<Xiān Zhào>

降钙素（**Calcitonin**）
<Jiàng Gài Sù>

麦角生物碱（**Ergot alkaloids**）
<Mài Jiǎo Shēng Wù Jiǎn>

偏瘫（**Hemiplegic**）
<Piān Tān>

偏头痛（**Migraine**）
<Piān Tóu Tòng>

恶心（**Nausea**）
<Ě Xīn>

恐声症（**Phonophobia**）
<Kǒng Shēng Zhèng>

畏光（**Photophobia**）
<Wèi Guāng>

瑞美吉泮（**Rimegepant**）
<Ruì Měi Jí Pàn>

## 更多信息

**欲了解更多信息，请点击此处查阅此研究的科研文章：**

**查看科研文章**

**如需了解有关各方面临床研究的更多信息，请访问：**

https://www.clinicaltrials.gov/ct2/about-studies/learn

https://www.cancerresearchuk.org/about-cancer/find-a-clinical-trial/what-clinical-trials-are

# 研究结果

## (瑞美吉泮或安慰剂)治疗的效果如何?

- 在服用瑞美吉泮或安慰剂2小时后，服用瑞美吉泮中有更多的人：
  - 摆脱疼痛（这意味着他们没有感到疼痛）。
  - 摆脱其他最困扰的偏头痛症状（这意味着除了疼痛之外，在接受治疗前最令他们困扰的症状已经消失）。
  - 疼痛缓解（这意味着他们没有感到疼痛或只有轻微疼痛）。
  - 功能正常（这意味着他们可以正常进行日常活动）。

在偏头痛发作并伴有中度或重度疼痛时，受试者服用一片瑞美吉泮或安慰剂。

受试者在接受治疗前和治疗后 2 小时对自己的疼痛程度、是否出现其他最困扰的症状以及活动能力进行评分。

| | 摆脱疼痛<br>（没有感到疼痛）受试者比例 | 摆脱最困扰的症状<br>（症状消失）的受试者比例 | 疼痛缓解<br>（无痛或轻度疼痛）的受试者比例 | 功能正常<br>（可以正常进行日常活动）的受试者比例 |
|---|---|---|---|---|
| 服用**瑞美吉泮**的人 | 18%<br>98 of 537 | 48%<br>258 of 537 | 65%<br>351 of 537 | 39%<br>176 of 457 |
| 服用**安慰剂**的人 | 11%<br>57 of 537 | 32%<br>171 of 537 | 48%<br>256 of 537 | 24%<br>110 of 462 |

对乙酰氨基酚（**Acetaminophen**）
<Duì Yǐ Xiān Ān Jī Fēn>

止吐药（**Antiemetics**）
<Zhǐ Tù Yào>

抗炎药（**Anti-inflammatory**）
<Kàng Yán Yào>

先兆
<Xiān Zhào>

降钙素（**Calcitonin**）
<Jiàng Gài Sù>

麦角生物碱（**Ergot alkaloids**）
<Mài Jiǎo Shēng Wù Jiǎn>

偏瘫（**Hemiplegic**）
<Piān Tān>

偏头痛（**Migraine**）
<Piān Tóu Tòng>

恶心（**Nausea**）
<Ě Xīn>

恐声症（**Phonophobia**）
<Kǒng Shēng Zhèng>

畏光（**Photophobia**）
<Wèi Guāng>

瑞美吉泮（**Rimegepant**）
<Ruì Měi Jí Pàn>

## 更多信息

**欲了解更多信息，请点击此处查阅此研究的科研文章：**

**查看科研文章**

**如需了解有关各方面临床研究的更多信息，请访问：**

https://www.clinicaltrials.gov/ct2/about-studies/learn

https://www.cancerresearchuk.org/about-cancer/find-a-clinical-trial/what-clinical-trials-are

对乙酰氨基酚（**Acetaminophen**）
<Duì Yǐ Xiān Ān Jī Fēn>

止吐药（**Antiemetics**）
<Zhǐ Tù Yào>

抗炎药（**Anti-inflammatory**）
<Kàng Yán Yào>

先兆
<Xiān Zhào>

降钙素（**Calcitonin**）
<Jiàng Gài Sù>

麦角生物碱（**Ergot alkaloids**）
<Mài Jiǎo Shēng Wù Jiǎn>

偏瘫（**Hemiplegic**）
<Piān Tān>

偏头痛（**Migraine**）
<Piān Tóu Tòng>

恶心（**Nausea**）
<Ě Xīn>

恐声症（**Phonophobia**）
<Kǒng Shēng Zhèng>

畏光（**Photophobia**）
<Wèi Guāng>

瑞美吉泮（**Rimegepant**）
<Ruì Měi Jí Pàn>

## 更多信息

**欲了解更多信息，请点击此处查阅此研究的科研文章：**

**查看科研文章**

**如需了解有关各方面临床研究的更多信息，请访问：**

https://www.clinicaltrials.gov/ct2/about-studies/learn

https://www.cancerresearchuk.org/about-cancer/find-a-clinical-trial/what-clinical-trials-are

- 与服用安慰剂的人相比，更多服用瑞美吉泮的人在用药后的2至24小时内持续摆脱了疼痛。
  - 这意味着他们在服用瑞美吉泮或安慰剂后的2小时到服用瑞美吉泮或安慰剂后的1天之内的任何时间都没有感到疼痛。
- 与服用安慰剂的人相比，更多服用瑞美吉泮的人在用药后的 2 至 48 小时内持续摆脱了疼痛。
  - 这意味着他们在服用瑞美吉泮或安慰剂后的2小时到服用瑞美吉泮或安慰剂后 的2 天之内的任何时间都没有感到疼痛。

在偏头痛发作并伴有中度或重度疼痛时，受试者服用一片瑞美吉泮或安慰剂。

受试者在接受治疗前以及治疗后 2 小时、3 小时、4 小时、6 小时、8 小时、24 小时（1 天）和 48 小时（2 天）时评估自己的疼痛程度。

**2 至 24 小时内持续无疼痛**的人的比例（从服用瑞美吉泮或安慰剂后的 2 小时到服用瑞美吉泮或安慰剂后的 1 天之内的任何时间都没有疼痛）

15%
82 of 537

服用**瑞美吉泮**的人

8%
43 of 537

服用**安慰剂**的人

**2 至 48小时内持续无疼痛**的人的比例（从服用瑞美吉泮或安慰剂后的 2 小时到服用瑞美吉泮或安慰剂后的 2 天之内的任何时间都没有疼痛）

15%
78 of 537

服用**瑞美吉泮**的人

7%
39 of 537

服用**安慰剂**的人

- 与服用安慰剂的人相比，服用瑞美吉的人在服药后的24小时（1 天）内需要使用其他药物（补救药物）治疗偏头痛的人数更少。

在偏头痛发作并伴有中度或重度疼痛时，受试者服用一片瑞美吉泮或安慰剂。

受试者记录了在接受治疗后的 24 小时（1 天）内使用的其他治疗偏头痛的药物。

在服用**瑞美吉泮或安慰剂后的 24 小时（1 天）内使用其他偏头痛药物（补救药物）**的比例

5%
28 of 537

服用**瑞美吉泮**的人

14%
75 of 537

服用**安慰剂**的人

## 治疗（瑞美吉泮或安慰剂）的安全性如何？

- 服用瑞美吉泮和服用安慰剂出现副作用的人数相似。
  – 副作用是指开始接受治疗后出现或加重的症状或病症。副作用可能与治疗有关，也可能无关。
  – 在研究过程中，服用瑞美吉泮或安慰剂的人最常见的副作用是感到胃部不适（也称为 “恶心” ）。

在偏头痛发作并伴有中度或重度疼痛时，受试者服用一片瑞美吉泮或安慰剂。

受试者记录了他们在接受治疗后出现的副作用

| 服用瑞美吉泮或安慰剂后**出现副作用**的比例 | 服用瑞美吉泮或安慰剂后**感到胃部不适（恶心）**的人数比例 |
|---|---|
| 15%<br>82 of 538<br>服用**瑞美吉泮**的人 | 1%<br>6 of 538<br>服用**瑞美吉泮**的人 |
| 16%<br>88 of 537<br>服用**安慰剂**的人 | 3%<br>14 of 537<br>服用**安慰剂**的人 |

- 在服用瑞美吉泮（538 人中有 1 人）或安慰剂（537 人中有 2 人）的人中，只有不到 1%的人出现了研究人员认为严重的副作用。
  – 当副作用危及生命、需要住院治疗、导致持久性问题或需要药物或手术治疗时，通常会被认为是严重的副作用。

- 更多研究结果请点击此处：查看科研文章

对乙酰氨基酚（**Acetaminophen**）
<Duì Yǐ Xiān Ān Jī Fēn>

止吐药（**Antiemetics**）
<Zhǐ Tù Yào>

抗炎药（**Anti-inflammatory**）
<Kàng Yán Yào>

先兆
<Xiān Zhào>

降钙素（**Calcitonin**）
<Jiàng Gài Sù>

麦角生物碱（**Ergot alkaloids**）
<Mài Jiǎo Shēng Wù Jiǎn>

偏瘫（**Hemiplegic**）
<Piān Tān>

偏头痛（**Migraine**）
<Piān Tóu Tòng>

恶心（**Nausea**）
<Ě Xīn>

恐声症（**Phonophobia**）
<Kǒng Shēng Zhèng>

畏光（**Photophobia**）
<Wèi Guāng>

瑞美吉泮（**Rimegepant**）
<Ruì Měi Jí Pàn>

## 更多信息

**欲了解更多信息，请点击此处查阅此研究的科研文章：**

查看科研文章

**如需了解有关各方面临床研究的更多信息，请访问：**

https://www.clinicaltrials.gov/ct2/about-studies/learn

https://www.cancerresearchuk.org/about-cancer/find-a-clinical-trial/what-clinical-trials-are

对乙酰氨基酚（**Acetaminophen**）
<Duì Yǐ Xiān Ān Jī Fēn>

止吐药（**Antiemetics**）
<Zhǐ Tù Yào>

抗炎药（**Anti-inflammatory**）
<Kàng Yán Yào>

先兆
<Xiān Zhào>

降钙素（**Calcitonin**）
<Jiàng Gài Sù>

麦角生物碱（**Ergot alkaloids**）
<Mài Jiǎo Shēng Wù Jiǎn>

偏瘫（**Hemiplegic**）
<Piān Tān>

偏头痛（**Migraine**）
<Piān Tóu Tòng>

恶心（**Nausea**）
<Ě Xīn>

恐声症（**Phonophobia**）
<Kǒng Shēng Zhèng>

畏光（**Photophobia**）
<Wèi Guāng>

瑞美吉泮（**Rimegepant**）
<Ruì Měi Jí Pàn>

## 更多信息

**欲了解更多信息，请点击此处查阅此研究的科研文章：**

查看科研文章

**如需了解有关各方面临床研究的更多信息，请访问：**

https://www.clinicaltrials.gov/ct2/about-studies/learn

https://www.cancerresearchuk.org/about-cancer/find-a-clinical-trial/what-clinical-trials-are

## 研究人员报告的主要结论是什么？

- 在这项研究中，与服用安慰剂的人相比，更多的人在服用瑞美吉泮两小时后就摆脱了疼痛和其他最困扰的偏头痛症状。
- 服用瑞美吉泮和服用安慰剂的人群出现的副作用相似。
- 这些结果表明，瑞美吉泮可能是治疗中国成年人偏头痛发作的有效新药。
- 但是，这项研究只考察了单剂量瑞美吉泮的效果。
  - 本研究无法就长期反复使用瑞美吉泮得出结论。

**是否有计划开展更多研究？**

有计划对瑞美吉泮开展更多研究。这些研究将有助于研究人员进一步了解瑞美吉泮治疗中国偏头痛患者的长期疗效和安全性。

**我在哪里可以找到更多信息？**

这项研究调查了中国或韩国居民使用瑞美吉泮治疗偏头痛的情况。这里概述的文章介绍了与中国居民相关的结果。所有受试者（中国和韩国）的结果均已公布。您可以点击此处访问之前的文章：
查看之前的科研文章。

有关本研究的更多信息，请访问：
**https://www.clinicaltrials.gov/study/NCT04574362**

如需了解有关各方面临床研究的更多信息，请访问：
**https://www.clinicaltrials.gov/ct2/about-studies/learn**

## 这项研究的申办者是谁？

本研究由 Biohaven Pharmaceuticals 的全资子公司 BioShin 申办。
2022 年 10 月，Biohaven 制药公司被辉瑞公司收购。
Pfizer Inc.
235 East 42nd Street NY, NY 10017
Phone (United States): +1 212-733-232
辉瑞公司对参与这项研究的所有人表示感谢。

**致谢**
本摘要由 Envision Pharma Group 的 Matt Soulsby 博士（CMPP）提供写作支持，并由辉瑞公司资助。完整文章的作者参与了本摘要的编写。
