# Supplementary material for: Rimegepant orally disintegrating tablet 75 mg for acute treatment of migraine in adults from China: a subgroup analysis of a double-blind, randomized, placebo-controlled, phase 3 clinical trial
Source: J Headache Pain. 2024 Apr 16;25(1):57. doi: 10.1186/s10194-024-01731-4 (PMC11020209; doi:10.1186/s10194-024-01731-4)
Supplement: Supplementary file 4 — Additional file 4. Chinese full manuscript. [file 10194_2024_1731_MOESM4_ESM.pdf]

瑞美吉泮口腔崩解片（75 毫克）急性治疗中国成人偏头痛：一项双盲、随机、安慰剂对照 3 期临床试验的亚组分析

于生元[1], 郭爱红[2], 王振[3], 刘建光[4], 谭戈[5], 杨谦[6], 张明洁[1], 哈斯也提·依不来音[7], 陈会生[8], 张拥波[9], Robert Croop[10], 孙艳慧[11]、 刘羽[12]、赵倩[13]、陆志红[11]

[1]中国人民解放军总医院，中国北京； [2]延安大学咸阳医院，中国咸阳； [3]长沙市中心医院，中国长沙； [4]武汉市第三医院，中国武汉； [5]重庆医科大学第一附属医院，中国重庆； [6]陕西省人民医院，中国西安； [7]新疆医科大学第二附属医院，中国新疆省乌鲁木齐； [8]中国人民解放军北部战区总医院，中国沈阳； [9]首都医科大学附属北京友谊医院，中国北京； [10]Biohaven Pharmaceuticals，美国康涅狄格州纽黑文； [11]辉瑞（中国）研究开发有限公司，中国上海； [12]辉瑞公司，中国北京； [13]辉瑞公司，中国成都。

通讯作者

陆志红，MD，PhD

辉瑞（中国）研究开发有限公司

上海浦东新区

纳贤路 60 号腾飞莲花商务园 3 号楼，邮编 201203

电子邮件： luzhihong57@outlook.com

摘要

背景：瑞美吉泮（Rimegepant）口腔崩解片（ODT）是一种口服小分子降钙素基因相关肽受体拮抗剂，在美国和其他国家适用于偏头痛的急性和预防性治疗。此前，一项大型临床试验评估了瑞美吉泮 ODT 75 mg 用于中国或韩国成人偏头痛急性治疗的有效性和安全性。本文对该试验进行事后亚组分析，评估瑞美吉泮对中国成年人偏头痛急性治疗的有效性和安全性。

研究方法： 符合条件的受试者年龄≥18 岁，偏头痛病史≥1 年，中度或重度疼痛发作 2 至 8 次/月，筛选前 3 个月内每月头痛 < 15 天。受试者自行使用瑞美吉泮 ODT 75 mg 或相应的安慰剂来治疗单次中度或重度疼痛强度的偏头痛发作。共同主要终点是给药后 2 小时无痛和无最令人困扰的症状 (MBS)。关键次要终点包括给药后 2 小时疼痛缓解、给药后 2 小时功能障碍恢复正常、给药后 24 小时挽救药物的使用情况以及给药后 2-24 小时和 2-48 小时的持续无疼痛率。所有 p 值均为名义值。通过治疗期间不良事件（TEAE）、心电图、生命体征和常规实验检查来评估安全性。

结果： 总体而言，1,075 名受试者（瑞美吉泮， $n = 538$；安慰剂， $n = 537$）被纳入亚组分析。瑞美吉泮与安慰剂相比，在无痛（18.2% vs 10.6%，$p = 0.0004$）和无 MBS（48.0% vs 31.8%，$p < 0.0001$）这两个共同主要终点以及所有关键次要终点上都具有更优疗效。瑞美吉泮组（15.2%）和安慰剂组（16.4%）的治疗期间不良事件（TEAE）发生率相当。在瑞美吉泮组中没有观察到药物引起的肝损伤信号，也没有报告与研究药物相关的严重 TEAE。

结论： 单剂量瑞美吉泮 75 mg 对于中国成人偏头痛的急性治疗有效，其安全性和耐受性与安慰剂相似。

临床试验注册： ClinicalTrials.gov NCT04574362。

关键词

瑞美吉泮；偏头痛；急性治疗；中国；临床试验

图文摘要

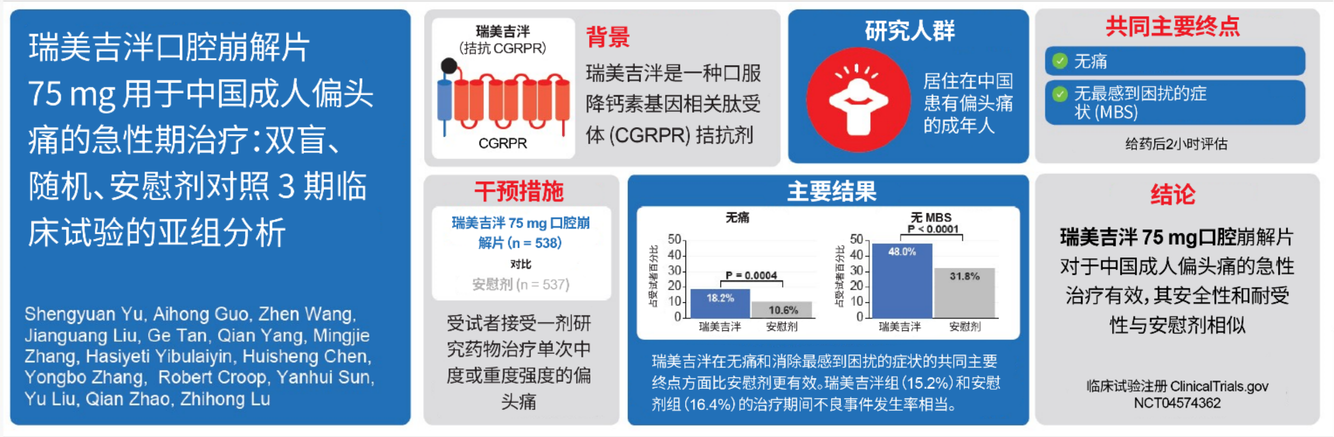

背景

偏头痛的临床特征为发作性、搏动性、中度至重度头痛，常伴有畏光、畏声、恶心或呕吐等症状[1]。偏头痛是全球最常见的失能性疾病之一，会对患者的日常活动、社交、生产力和整体生活质量产生负面影响[2-9]。尽管偏头痛是一个全球性的问题，但由于教育和资源、获得治疗的机会、治疗费用和其他社会经济因素的差异，偏头痛护理的质量在不同国家之间是存在差异的[10, 11]。

受偏头痛影响的中国人约有1.516亿[3]。然而，偏头痛在中国普遍未被充分认识，关键问题之一是误诊[12, 13]。在中国，大约一半的偏头痛患者不会就医，而是自行使用阿司匹林和非甾体抗炎药 (NSAID) 等非处方药来治疗偏头痛发作[14, 15]。而去医院就诊的患者通常会接受初级保健医生而非神经科医生/专科医生的诊治，且中国头痛中心的数量极少[15, 16]。尽管中国的偏头痛治疗指南与西方国家基本一致，但偏头痛预防的理念在中国采纳情况不佳，预防药物的使用率很低，即使在头痛门诊也是如此。[12, 13, 16]。这可能与中国偏头痛总体诊断率较低、以及大多数预防性治疗手段在中国并未正式获批（或报销）预防治疗的适应症有关。

中国偏头痛诊治指南推荐非甾体抗炎药、对乙酰氨基酚、含咖啡因的复合制剂、曲坦类、拉米地坦（lasmiditan）、瑞美吉泮和乌布吉泮（ubrogepant）用于偏头痛发作的急性期治疗[17]。一项基于中国成人偏头痛受试者的医疗保险理赔的回顾性分析（ *n* = 10,652）发现，只有26.4%（ *n* = 2,813) 的受试者接受了治疗偏头痛或缓解疼痛的急性期治疗药物[16]。在 2,813 名受试者中，最常用的处方药物是非阿司匹林的 NSAID (68.8%), 其次分别是阿司匹林 (8.0%)、阿片类药物 (7.1%)、麦角生物碱 (6.1%)和对乙酰氨基酚 (4.3%)[16]。值得注意的是，只有 3.3% 的受试者使用了曲坦类药物。中国（和其他国家）的指南推荐将曲坦类药物用于偏头痛的急性期治疗。曲坦类药物在美国和欧盟也是常用的急性期治疗药物[16, 18-24]。与西方国家不同的是，中成药和草药在中国也常用来治疗偏头痛[16, 25]。

上述一些急性疗法是具有局限性的。例如，患有某些心血管疾病的受试者禁用非甾体抗炎药和曲坦类；或有心血管风险因素的受试者慎用非甾体抗炎药和曲坦类[26, 27]。此外，非甾体抗炎药、曲坦类

药物、麦角生物碱和复方镇痛药与药物过度使用性头痛 (MOH) 的发生风险相关[1, 28]。MOH 在中国是一个令人担忧的问题，因为它对患者的生活质量有显著影响，经常无法确诊，或被误认为是偏头痛的恶化[29]。在一项对中国一家头痛中心的 1,453 名成年偏头痛患者的回顾性分析中，6.5%（n = 240）的患者符合MOH的诊断标准[29]。此外，许多患者对现有急性治疗方案的反应不佳；中国有研究预估，超过40%的患者对急性治疗反应不足[8, 30]。反应不足可能导致对治疗的不满意，同时也是发作性偏头痛转变为慢性偏头痛的风险因素[31]。因此，中国对安全有效的偏头痛急性治疗药物的需求显然尚未得到满足[8, 30, 32]。

降钙素基因相关肽 (CGRP) 在偏头痛的病理生理学中发挥着关键作用，美国和欧盟已批准多种针对 CGRP 信号传导的药物用于偏头痛治疗[33]。然而，尽管中国指南推荐 CGRP 受体拮抗剂瑞美吉泮和乌布吉泮作为偏头痛急性治疗药物，但乌布吉泮尚未在中国获批，目前仅瑞美吉泮近期（2024年1月）在中国获批，但也还未进入国家医保目录 [17, 34]。瑞美吉泮在美国、欧盟和英国均批准用于偏头痛的急性治疗和发作性偏头痛的预防性治疗[35, 36]。在美国进行的三项关键性、随机、安慰剂对照、3 期临床试验和一项开放标签、长期（1 年）、2/3 期安全性试验中，初步确立了瑞美吉泮用于偏头痛急性期治疗的有效性和安全性[37-40]。随后进行的一项随机、安慰剂对照的 3 期试验显示，瑞美吉泮75 毫克口腔崩解片 (ODT) 用于中国或韩国成人的偏头痛急性治疗是有效且安全的（NCT04574362），同时，该试验也是首个在美国以外的国家开展的瑞美吉泮用于偏头痛急性治疗的临床试验[41]。在中国和韩国成人偏头痛受试者中，瑞美吉泮在所有主要和关键次要疗效终点上均优于安慰剂，且安全性和耐受性与安慰剂相当[41]。

由于人口统计学、偏头痛治疗方法/可及性以及其他社会或文化因素的不同，韩国和中国受试者对瑞美吉泮的反应可能会有所不同。此外，目前尚缺乏瑞美吉泮用于中国成人偏头痛急性治疗的特异性数据。因此，我们进行了该亚组分析（基于之前在韩国和中国受试者中进行的试验 NCT04574362），以评估瑞美吉泮 ODT 75 mg 对中国成人偏头痛患者急性治疗的疗效和安全性。

方法

研究设计和资格标准

完整的研究方法和伦理监督细节之前已发表[41]。简而言之，该研究（NCT04574362）包括 3-28 天的筛选期、最长 45 天的急性治疗期或直到受试者出现中度或重度强度的偏头痛发作，以及用药后 7 天内的治疗结束访视（**图 1**）。

主要纳入标准包括：年龄≥18 岁；根据《国际头痛疾病分类》第三版（测试版）[42]，受试者患有符合偏头痛诊断的头痛（有或无先兆）至少 1 年；每月发生 2 至 8 次中度或重度强度的偏头痛，在不接受治疗的情况下，偏头痛发作的平均持续时间约为 4-72 小时，筛查前 3 个月每月头痛天数小于 15 天。排除有脑干先兆偏头痛或偏瘫性偏头痛病史的受试者。有曲坦类药物禁忌症的受试者如果符合其他所有研究入选标准，则纳入研究。接受预防性偏头痛药物治疗的受试者可以继续接受预防性治疗，前提是在筛选前剂量稳定 ≥ 3 个月。排除之前参加过其他试验性 CGRP 拮抗剂（小分子或生物制剂）相关研究或在过去 6 个月内使用过 CGRP 抗体的受试者。

治疗

通过交互式网络应答系统，将符合资格标准的受试者以 1:1 的比例随机分配给瑞美吉泮 ODT 75 毫克组或相应的安慰剂治疗组。按国家（中国和韩国）和预防药物的使用（是与否）对随机化进行分层。随机分组后，受试者被分发一剂试验用药，并要求在 45 天内治疗中度或重度强度的偏头痛发作（自行用药）。

使用试验药物后的 2 小时内，受试者禁止使用其他所有的头痛药物。如果受试者在给药 2 小时后疼痛仍未缓解（或在给药 2 小时后疼痛虽然缓解，但 2-48 小时期间，疼痛强度又恢复到中度或重度），则允许其使用以下挽救药物：阿司匹林、布洛芬、对乙酰氨基酚（最高 1000 毫克/天）、非甾体抗炎药、止吐药或巴氯芬。如果需要，受试者可以在使用试验药物的 48 小时后接受标准的偏头痛治疗。受试者在纸质日记中记录挽救药物的使用情况。

疗效评估

受试者在电子日记中记录疗效数据，包括头痛发作的时间、疼痛强度、相关偏头痛症状是否存在以及功能障碍的评级。采用 4 分制数字评分量表来评估疼痛的严重程度（0 = 无, 1 = 轻度, 2 = 中度，3 = 重度）和功能障碍程度（0 =功能正常，1 = 轻度受损，2 = 重度受损，3 = 需要卧床）。对偏头痛相关症状（恶心、畏光和畏声）的情况采用二元量表进行评估（0 = 未出现，1 = 出现），并要求受试者在用药前，从恶

心、畏光和畏声中选出最令人困扰的症状（MBS）。受试者需在用药前以及用药后的 15 分钟、30 分钟、45 分钟、60 分钟、90 分钟、2 小时、3 小时、4 小时、6 小时、8 小时、24 小时和 48 小时，分别对疼痛严重程度、相关偏头痛症状和功能障碍进行评估。

无痛和 无 MBS 定义为相应数字评分量表上的 0 分。疼痛缓解定义为疼痛评分量表上的 0 或 1 分。

疗效终点

共同主要终点是给药 2 小时后无痛的受试者比例以及给药 2 小时后无 MBS 的受试者比例。关键次要终点包括：给药 2 小时后疼痛缓解的受试者比例、给药 2 小时后功能恢复正常的受试者比例（在使用试验药物前有任何程度功能受损的受试者中）、给药后 24 小时内使用挽救药物的受试者比例、给药后 2-24 小时持续无痛的受试者比例以及给药后 2-48 小时持续无痛的受试者比例。

其他次要或探索性终点包括给药后 15 分钟、30 分钟、45 分钟、60 分钟、90 分钟、3 小时、4 小时、6 小时、8 小时、24 小时和 48 小时无痛、无 MBS、疼痛缓解和功能恢复正常的受试者比例。在给药后 2 小时实现无痛的受试者中，还评估了在给药后 2-48 小时内任何时间点疼痛复发（疼痛严重程度评分为 1、2 或 3 分）的比例。

安全性评估

通过治疗期间不良事件（TEAE）、心电图（ECG）、生命体征、体格检查和常规实验室检查来评估安全性。TEAE 的定义是试验用药品治疗时或治疗之后发生的治疗中发生的不良事件. 本研究对 TEAE 进行了描述性总结，其严重程度及与研究药物的关系将由现场研究人员确定。采用国际医学用语词典（MedDRA 23.0 版）对所有 TEAE 进行编码。

统计考虑因素

本文的所有分析都是在中国受试者亚组中进行的。统计分析计划中预先指定了该亚组的共同主要终点和关键次要疗效终点。该亚组中其他次要疗效终点的分析则是事后进行。在所有接受了试验用药品治疗、治疗时有中度或重度强度的偏头痛且提供了至少一个治疗后有效性数据点的随机化中国受试者中进行疗效分析。所有接受试验用药品治疗的中国受试者都经过了安全性评估。

采用 Cochran-Mantel-Haenszel 检验对瑞美吉泮与安慰剂的共同主要疗效终点和次要疗效终点进行比较，根据偏头痛预防药物的使用情况进行分层，双侧 α 水平为 0.05。中国亚组的统计分析未控制 1 型错误，所有 *p* 值均为名义值。对于无痛、无 MBS、疼痛缓解和功能恢复正常等终点，如果评估时间点的数据缺失，或者受试者在评估时间点或之前使用了挽救药物，则受试者的数据将被归为失败数据（在无 MBS 的分析中，受试者在偏头痛发作时未报告 MBS 的数据也将被归为失败数据）。对于 2-24 小时内持续无痛这一终点，如果在第 2 或 24 小时数据缺失，或在超过 1 个其他时间点（3、4、6 或 8 小时）数据缺失，或在 24 小时或之前使用过挽救药物，则受试者的数据将被归为失败数据。对于 2-48 小时内持续无痛

这一终点，如果在第 2、24、或 48 小时数据缺失，或在超过 1 个其他时间点（3、4、6 或 8 小时）数据缺失，或在 48 小时或之前使用过挽救药物，则受试者的数据将被归为失败数据。对于疼痛复发这一终点，如果受试者在第 24 或 48 小时数据缺失、在超过 1 个时间点上数据缺失，或者在 2-48 小时内使用了挽救药物，则受试者的数据将被归为失败（即疼痛复发）数据。使用 SAS 9.4 版（Cary, NC, USA）进行所有统计分析。

结果

受试者

共有 1,075 名中国受试者（瑞美吉泮 n = 538；安慰剂 n = 537) 接受试验药品治疗。所有接受治疗的中国受试者的人口统计学和临床特征如**表 1** 所示。受试者的中位年龄 35 岁（范围从 18 岁-71 岁），大多数人 (79.0%) 为女性。每月中度或重度偏头痛发作的中位（范围）次数为 3 次 (2-8)，最常报告的 MBS 史为恶心 (52.0%)。3.5% 的中国研究人群使用了预防性偏头痛药物。

疗效

共同主要终点发现，在给药后 2 小时无痛（瑞美吉泮 18.2% vs. 安慰剂 10.6%; 风险差异 = 7.6；p = 0.0004）和无 MBS（48.0% vs. 31.8%; 风险差异 = 16.2；p < 0.0001）方面，瑞美吉泮都显著优于安慰剂（**表 2**）。

瑞美吉泮在所有关键次要终点上也比安慰剂更有效（**表 2**），包括给药后 2 小时的疼痛缓解率（65.4% vs. 47.7%; $p < 0.0001$），给药后 2 小时功能恢复正常的比例（38.5% vs. 23.8%; $p < 0.0001$），给药后 24 小时内使用挽救药物的比例（5.2% vs. 14.0%; $p < 0.0001$），给药后 2-24 小时的持续无痛率（15.3% vs. 8.0%; $p = 0.0002$），以及给药后 2-48 小时内的持续无痛率（14.5% vs. 7.3%; $p = 0.0001$）。

给药后 15 分钟至 48 小时的无痛、无 MBS 、疼痛缓解和功能恢复正常的时间过程如**图 2** 和**补充表 1** 所示。瑞美吉泮相比安慰剂（$p < 0.05$），最早可在给药后 90 分钟即可实现无痛，60 分钟即可实现无 MBS，45 分钟即可实现缓解疼痛，60 分钟即可恢复正常功能。针对上述每个终点，瑞美吉泮相对安慰剂的优势（$p < 0.05$）在用药后 48 小时内的所有时间点均得以保持。在给药后 2 小时无痛的受试者中，瑞美吉泮组 20.4% 的患者在 48 小时内疼痛复发，安慰剂组中这一比例为 31.6%（$p = 0.1382$）。

安全性

瑞美吉泮组的 TEAE 总体发生率为 15.2%，安慰剂组为 16.4%（**表 3**）。各治疗组之间最常报告的 TEAE（≥ 0.5%的瑞美吉泮受试者报告）发生率相当。在这些 TEAE 中，仅蛋白尿检出（瑞美吉泮，1.5%；安慰剂，1.3%）和恶心（瑞美吉泮，1.1%；安慰剂，2.6%）的瑞美吉泮组报告人数≥1%。

瑞美吉泮组和安慰剂组分别有 1 人（0.2%）和 2 人（0.4%）发生严重 TEAE。瑞美吉泮组没有出现药物相关的严重 TEAE，安慰剂组有 1 例（0.2%）。瑞美吉泮治疗组药物相关的 TEAE 总发生率为 8.4%，安慰剂为 8.0%。

两个治疗组均无死亡病例报告，心电图、生命体征、体格检查结果或常规实验室检查均未出现有临床意义的变化。没有受试者出现丙氨酸转氨酶或天门冬氨酸氨基转移酶浓度大于正常值上限 3 倍、总胆红素浓度大于正常值上限 2 倍的情况。

讨论

之前的一项随机、安慰剂对照 3 期试验证明，瑞美吉泮 ODT 75 毫克用于中国或韩国成人偏头痛急性治疗有效且安全，并证实美国人群的研究结果可推广至中国和韩国人群。 [41] 。然而，仍缺少关于瑞美吉泮用于中国成人偏头痛急性治疗的特异性数据。因此，我们基于之前在韩国和中国受试者中进行的试验，进行了亚组分析，以评估瑞美吉泮 ODT 75 mg 对于居住在中国的成人偏头痛患者的疗效和安全性。在这一中国成人亚组中，瑞美吉泮在所有共同主要疗效终点和关键次要疗效终点上的疗效均优于安慰剂，其 TEAE 情况与安慰剂相当。这些亚组结果与在总体研究人群中观察到的结果一致[41]，并证实瑞美吉泮对中国成人偏头痛的急性治疗是有效的。

具体来看，在这项研究的中国受试者中，瑞美吉泮与安慰剂相比，在用药后 2 小时无痛和无 MBS 这两个共同主要疗效终点上都有所改善。与安慰剂相比，瑞美吉泮在 2 小时疼痛缓解和功能恢复正常这两项终点指标上也有所改善。此外，与安慰剂组相比，瑞美吉泮组在用药后 24 小时内需要使用挽救药物的人数更少。与安慰剂相比，在用药后 45 分钟疼痛缓解终点、60 分钟功能恢复正常终点和无 MBS 终点以及 90 分钟无痛终点均有所改善。在用药后 48 小时内的所有后续评估中，都能观察到这些改善，包括

无痛、无 MBS、疼痛缓解和功能恢复正常，这表明瑞美吉泮对许多受试者具有持续的疗效。此外，与安慰剂组相比，瑞美吉泮组在给药后 2-24 小时和 2-48 小时内持续无痛的人数比例更高，这一观察结果也进一步证实了瑞美吉泮的持续疗效。总体而言，在中国受试者中的研究结果与之前在美国进行的瑞美吉泮用于偏头痛急性治疗的研究结果是一致的，在这些研究中，用药后 2 小时的无痛、无 MBS 、疼痛缓解和功能恢复正常，用药后 24 小时的挽救药物使用，以及用药后 2-24 小时和 2-48 小时的持续无痛都相对安慰剂有所改善[37, 38, 40]。

因为瑞美吉泮的总体安全性与安慰剂相当，因此在中国受试者身上观察到的安全性结果也与之前在美国进行的瑞美吉泮试验结果一致。 [37, 38、40]。在中国受试者亚组中，瑞美吉泮组未出现与治疗相关的严重 TEAE，大多数 TEAE 的严重程度为轻度或中度，大多数 TEAE 无需治疗即可缓解，TEAE 在瑞美吉泮组和安慰剂组中的发生率相似，未观察到心电图或常规实验室检查有临床意义的变化，也未出现肝毒性信号。

本亚组分析显示瑞美吉泮具有良好的疗效和安全性，这表明瑞美吉泮可能有助于满足中国对安全有效的偏头痛急性治疗药物的需求。中国偏头痛诊治指南推荐非甾体抗炎药用于偏头痛的急性治疗，这类药物也是在中国最常用的偏头痛急性治疗药物[43]。然而，非甾体抗炎药可能无法有效治疗剧烈的偏头痛发作。[18, 44]此外，此类药物的长期应用与胃肠道出血、心血管血栓事件和肾损伤有关[45-47]。因此，患有胃肠道疾病、心血管疾病或肾功能不全或存在上述风险的患者应慎用此类药物[27]。中国偏头痛诊治指南也推荐曲坦类药物，但曲坛类药物在中国并不常用 [16, 43]。这可能是由于多种因素造成的，包

括成本、获取途径，以及曲坦类药物由于其血管收缩特性导致的心血管疾病患者禁忌[26]。非甾体抗炎药、曲坦类药物和其他急性偏头痛治疗药物还存在 MOH 风险[28]。相比之下，瑞美吉泮与其他吉泮类药物（如扎维吉泮（zavegepant）、乌布吉泮）一样，与 MOH 无关[48]。例如，与安慰剂相比，瑞美吉泮每隔一天给药用于偏头痛的预防性治疗，连续使用 12 周，可显著减少每月偏头痛的天数，同时没有出现 MOH。[49] 此外，有证据显示，使用瑞美吉泮治疗偏头痛既降低了 MOH 的时点患病率，也减少了患者对某些可导致 MOH 药物（包括巴比妥类药物和阿片类药物）的需求[50-52]。

尽管 MOH 的确切机制尚不清楚，但一般认为其涉及下行疼痛调节的变化、痛觉感受器的敏化、神经系统的其他结构和功能改变，以及可能的生物行为因素[53, 54]。在药物过度使用的临床前模型中，重复使用吉泮类药物与 MOH 的感觉变化无关[55, 56]。

虽然瑞美吉泮在本研究中显示出偏头痛急性治疗的疗效和良好的安全性，但本研究旨在评估单剂量瑞美吉泮的疗效，因此无法对中国成人反复长期使用瑞美吉泮的疗效或安全性下结论。其他正在进行的研究（NCT05371652、NCT05810038）将评估瑞美吉泮在中国人群中的长期使用情况。此外，对当前中国受试者亚组的统计分析未控制 1 型错误，所有 *p* 值均为名义值。

总体而言，本研究表明，单剂量瑞美吉泮 ODT 75 毫克对中国成年人偏头痛的急性治疗有效且耐受性良好。上述结果表明，使用瑞美吉泮可能有助于满足中国对安全有效的偏头痛急性期治疗的需求。这一结论得到了中国偏头痛诊断和治疗指南的支持，该指南基于多项随机、安慰剂对照临床试验所证实的瑞美吉泮良好的疗效和安全性的高级别证据，强烈建议将其用于偏头痛急性期治疗[17]。

缩略语表

CGRP	降钙素基因相关肽
ECG	心电图
MBS	最令人困扰的症状
MOH	药物过度使用性头痛
NSAID	非甾体类抗炎药
ODT	口腔崩解片
TEAE	治疗期间不良事件

声明

伦理声明和知情同意声明

本研究方案已获得各参与研究中心的机构审查委员会或独立伦理委员会的批准。所有参与者均提供了书面知情同意书。本研究按照《赫尔辛基宣言》和所有《国际协调会议良好临床实践指南》的伦理原则进行。

同意发表声明

不适用

数据和材料的可用性

如需要支持本研究结果的数据，辉瑞公司将在收到请求及审核后提供。根据某些标准、条件和例外情况，辉瑞还可提供对相关去标识化个人受试者数据的访问权限。进一步信息请访问 https://www.pfizer.com/science/clinical-trials/trial-data-and-results.

利益冲突

于生元、郭爱红、王振、刘建光、谭戈、杨谦、张明洁、哈斯也提·依不来音、陈会生和张拥波没有需要申报的竞争利益。Robert Croop 是 Biohaven Pharmaceuticals 的员工，拥有 Biohaven Ltd 的股票，曾是辉瑞公司的员工，从辉瑞公司领取研究费用，并为 Collima LLC 提供服务。Collima LLC 与辉瑞公司、Aptose Biosciences Inc.、Manistee Therapeutics 和 Vida Ventures Management Co.，L.L.C. 签订了咨询协议。孙艳慧、刘羽和赵倩是辉瑞公司的员工，拥有辉瑞公司的股票。陆志红曾是 Bioshin（Biohaven 子公司）的员工，目前是辉瑞公司的员工。

资助

本研究由 Biohaven Pharmaceuticals 的全资子公司 BioShin 赞助。Biohaven Pharmaceuticals 于 2022 年 10 月被辉瑞收购。Bioshin、Biohaven 和/或辉瑞公司参与了研究设计、数据收集、数据分析和数据解读。这些公司的员工以作者的身份参与了稿件的编写。

作者贡献

于生元、张明洁、RC 和陆志红参与了研究设计。于生元、郭爱红、王振、刘建光、谭戈、杨谦、张明洁、哈斯也提·依不来音、陈会生和张拥波登记了本次研究的受试者。陆志红监督了临床试验。孙艳慧参与统计分析。于生元和陆志红验证了数据。所有作者都参与了数据解读，编辑了稿件内容和提高其准确性。所有作者都可以完全访问研究中的所有 数据，并对提交稿件发表的决定负有最终责任。

致谢

医学写作支持由 Engage Scientific Solutions 的 Matt Soulsby 博士、CMPP 提供，并由辉瑞公司资助。作者感谢研究参与者、研究人员和研究中心工作人员。

参考文献

1. Headache Classification Committee of the International Headache Society (IHS) The International Classification of Headache Disorders, 3rd edition. (2018) Cephalalgia. 38:1-211. doi: 10.1177/0333102417738202.
2. Global, regional, and national incidence, prevalence, and years lived with disability for 328 diseases and injuries for 195 countries, 1990-2016: a systematic analysis for the Global Burden of Disease Study 2016. (2017) Lancet. 390:1211-1259. doi: 10.1016/s0140-6736(17)32154-2.

3. Yao C, Wang Y, Wang L, Liu Y, Liu J, Qi J, et al (2019) Burden of headache disorders in China, 1990-2017: findings from the Global Burden of Disease Study 2017. J Headache Pain. 20:102. doi: 10.1186/s10194-019-1048-2.

4. Abu Bakar N, Tanprawate S, Lambru G, Torkamani M, Jahanshahi M, Matharu M (2016) Quality of life in primary headache disorders: A review. Cephalalgia. 36:67-91. doi: 10.1177/0333102415580099.

5. Blumenfeld A, Varon S, Wilcox T, Buse D, Kawata A, Manack A, et al (2011) Disability, HRQoL and resource use among chronic and episodic migraineurs: Results from the International Burden of Migraine Study (IBMS). Cephalalgia. 31:301-315. doi: 10.1177/0333102410381145.

6. Shimizu T, Sakai F, Miyake H, Sone T, Sato M, Tanabe S, et al (2021) Disability, quality of life, productivity impairment and employer costs of migraine in the workplace. J Headache Pain. 22:29. doi: 10.1186/s10194-021-01243-5.

7. Buse DC, Nahas SJ, Stewart WBF, Armand CE, Reed ML, Fanning KM, et al (2023) Optimized Acute Treatment of Migraine Is Associated With Greater Productivity in People With Migraine: Results From the Chronic Migraine Epidemiology and Outcomes (CaMEO) Study. J Occup Environ Med. 65:e261-e268. doi: 10.1097/jom.0000000000002801.

8. Zhao H, Xiao Z, Zhang L, Ford J, Zhong S, Ye W, et al (2023) Real-World Treatment Patterns and Outcomes Among Patients with Episodic Migraine in China: Results from the Adelphi Migraine Disease Specific Programme™. J Pain Res. 16:357-371. doi: 10.2147/jpr.S371887.

9. Wang X, Xing Y, Sun J, Zhou H, Yu H, Zhao Y, et al (2016) Prevalence, Associated Factors, and Impact on Quality of Life of Migraine in a Community in Northeast China. J Oral Facial Pain Headache. 30:139-149. doi: 10.11607/ofph.1584.

10. Raffaelli B, Rubio-Beltrán E, Cho SJ, De Icco R, Labastida-Ramirez A, Onan D, et al (2023) Health equity, care access and quality in headache - part 2. J Headache Pain. 24:167. doi: 10.1186/s10194-023-01699-7.

11. Martelletti P, Leonardi M, Ashina M, Burstein R, Cho SJ, Charway-Felli A, et al (2023) Rethinking headache as a global public health case model for reaching the SDG 3 HEALTH by 2030. J Headache Pain. 24:140. doi: 10.1186/s10194-023-01666-2.

12. Li X, Zhou J, Tan G, Wang Y, Ran L, Chen L (2012) Diagnosis and treatment status of migraine: a clinic-based study in China. J Neurol Sci. 315:89-92. doi: 10.1016/j.jns.2011.11.021.

13. Liu H, Dong M, Liu K, Jia Z, Gui W, Cheng Y, et al (2023) Status of diagnosis and preventative treatment for primary headache disorders: real-world data of unmet needs in China. The Journal of Headache and Pain. 24:119. doi: 10.1186/s10194-023-01654-6.

14. Yu S, Zhang M, Zhou J, Liu R, Wan Q, Li Y (2014) Headache care in China. Headache. 54:601-609. doi: 10.1111/head.12330.

15. Liu R, Yu S, He M, Zhao G, Yang X, Qiao X, et al (2013) Health-care utilization for primary headache disorders in China: a population-based door-to-door survey. J Headache Pain. 14:47. doi: 10.1186/1129-2377-14-47.

16. Yu S, Zhang Y, Yao Y, Cao H (2020) Migraine treatment and healthcare costs: retrospective analysis of the China Health Insurance Research Association (CHIRA) database. The Journal of Headache and Pain. 21:53. doi: 10.1186/s10194-020-01117-2.

17. 中国偏头痛诊治指南（2022 年版）。 (2022)中华疼痛医学杂志。 28:881-898.

18. Ailani J, Burch RC, Robbins MS, Society tBoDotAH (2021) The American Headache Society Consensus Statement: Update on integrating new migraine treatments into clinical practice. Headache: The Journal of Head and Face Pain. 61:1021-1039. doi: https://doi.org/10.1111/head.14153.

19. Evers S, Afra J, Frese A, Goadsby PJ, Linde M, May A, et al (2009) EFNS guideline on the drug treatment of migraine--revised report of an EFNS task force. Eur J Neurol. 16:968-981. doi: 10.1111/j.1468-1331.2009.02748.x.

20. Marmura MJ, Silberstein SD, Schwedt TJ (2015) The acute treatment of migraine in adults: the american headache society evidence assessment of migraine pharmacotherapies. Headache. 55:3-20. doi: 10.1111/head.12499.
21. Hutchinson S, Lipton RB, Ailani J, Reed ML, Fanning KM, Adams AM, et al (2020) Characterization of Acute Prescription Migraine Medication Use: Results From the CaMEO Study. Mayo Clin Proc. 95:709-718. doi: 10.1016/j.mayocp.2019.11.025.
22. Lipton RB, Munjal S, Alam A, Buse DC, Fanning KM, Reed ML, et al (2018) Migraine in America Symptoms and Treatment (MAST) Study: Baseline Study Methods, Treatment Patterns, and Gender Differences. Headache. 58:1408-1426. doi: 10.1111/head.13407.
23. Roessler T, Zschocke J, Roehrig A, Friedrichs M, Friedel H, Katsarava Z (2020) Administrative prevalence and incidence, characteristics and prescription patterns of patients with migraine in Germany: a retrospective claims data analysis. J Headache Pain. 21:85. doi: 10.1186/s10194-020-01154-x.
24. Vo P, Paris N, Bilitou A, Valena T, Fang J, Naujoks C, et al (2018) Burden of Migraine in Europe Using Self-Reported Digital Diary Data from the Migraine Buddy© Application. Neurol Ther. 7:321-332. doi: 10.1007/s40120-018-0113-0.
25. Lyu S, Zhang CS, Sun J, Weng H, Xue CC, Guo X, et al (2022) Chinese herbal medicine for migraine management: A hospital-based retrospective analysis of electronic medical records. Front Med (Lausanne). 9:936234. doi: 10.3389/fmed.2022.936234.
26. Dodick DW, Shewale AS, Lipton RB, Baum SJ, Marcus SC, Silberstein SD, et al (2020) Migraine Patients With Cardiovascular Disease and Contraindications: An Analysis of Real-World Claims Data. J Prim Care Community Health. 11:2150132720963680. doi: 10.1177/2150132720963680.
27. Antman EM, Bennett JS, Daugherty A, Furberg C, Roberts H, Taubert KA (2007) Use of nonsteroidal antiinflammatory drugs: an update for clinicians: a scientific statement from the American Heart Association. Circulation. 115:1634-1642. doi: 10.1161/circulationaha.106.181424.

28. Cooper W, Doty EG, Hochstetler H, Hake A, Martin V (2020) The current state of acute treatment for migraine in adults in the United States. Postgrad Med. 132:581-589. doi: 10.1080/00325481.2020.1767402.

29. Dong Z, Chen X, Steiner TJ, Hou L, Di H, He M, et al (2015) Medication-overuse headache in China: Clinical profile, and an evaluation of the ICHD-3 beta diagnostic criteria. Cephalalgia. 35:644-651. doi: 10.1177/0333102414552533.

30. Zhang L, Novick D, Zhong S, Li J, Walker C, Harrison L, et al (2023) Real-World Analysis of Clinical Characteristics, Treatment Patterns, and Patient-Reported Outcomes of Insufficient Responders and Responders to Prescribed Acute Migraine Treatment in China. Pain and Therapy. doi: 10.1007/s40122-023-00494-1.

31. Lipton RB, Fanning KM, Serrano D, Reed ML, Cady R, Buse DC (2015) Ineffective acute treatment of episodic migraine is associated with new-onset chronic migraine. Neurology. 84:688-695. doi: 10.1212/wnl.0000000000001256.

32. Luo N, Qi W, Zhuang C, Di W, Lu Y, Huang Z, et al (2014) A Satisfaction Survey of Current Medicines Used for Migraine Therapy in China: Is Chinese Patent Medicine Effective Compared with Western Medicine for the Acute Treatment of Migraine? Pain Medicine. 15:320-328. doi: 10.1111/pme.12277.

33. Edvinsson L (2019) Role of CGRP in Migraine. Handb Exp Pharmacol. 255:121-130. doi: 10.1007/164_2018_201.

34. Monthly Report: New Drug Approvals in China, September 2023 (2023). https://baipharm.chemlinked.com/news/monthly-report-new-drug-approvals-in-china-september-2023. Accessed October 9 2023.

35. Nurtec ODT (rimegepant) Prescribing information. https://labeling.pfizer.com/ShowLabeling.aspx?id=19036。Accessed May 12 2023.

36. Vydura (Rimegepant) Prescribing Information. https://www.pfizerpro.co.uk/medicine/vydura. Accessed 2023 May 12.

37. Lipton RB, Croop R, Stock EG, Stock DA, Morris BA, Frost M, et al (2019) Rimegepant, an Oral Calcitonin Gene-Related Peptide Receptor Antagonist, for Migraine. N Engl J Med. 381:142-149. doi: 10.1056/NEJMoa1811090.

38. Croop R, Goadsby PJ, Stock DA, Conway CM, Forshaw M, Stock EG, et al (2019) Efficacy, safety, and tolerability of rimegepant orally disintegrating tablet for the acute treatment of migraine: a randomised, phase 3, double-blind, placebo-controlled trial. The Lancet. 394:737-745. doi: 10.1016/S0140-6736(19)31606-X.

39. Croop R, Berman G, Kudrow D, Mullin K, Thiry A, Lovegren M, et al (2023) A Multicenter, Open-Label Long-Term Safety Study of Rimegepant for the Acute Treatment of Migraine. Cephalalgia [Manuscript in Press].

40. Lipton RB, Conway CM, Stock EG, Stock D, Morris BA, McCormack TJ, et al (2018) Efficacy, Safety, and Tolerability of Rimegepant 75 mg, an Oral CGRP Receptor Antagonist, for the Acute Treatment of Migraine: Results from a Phase 3, Double-Blind, Randomized, Placebo-Controlled Trial, Study 301. Headache: The Journal of Head and Face Pain. 58:1287-1337.

41. Yu S, Kim B-K, Guo A, Kim M-H, Zhang M, Wang Z, et al (2023) Safety and efficacy of rimegepant orally disintegrating tablet for the acute treatment of migraine in China and South Korea: a phase 3, double-blind, randomised, placebo-controlled trial. The Lancet Neurology. 22:476-484. doi: 10.1016/S1474-4422(23)00126-6.

42. The International Classification of Headache Disorders, 3rd edition (beta version). (2013) Cephalalgia. 33:629-808. doi: 10.1177/0333102413485658.

43. Chinese Medical Association Group (2016) Guide to the prevention and treatment of migraine in China [Chinese]. . Chin J Pain Med 22:721–727.

44. Mayans L, Walling A (2018) Acute Migraine Headache: Treatment Strategies. Am Fam Physician. 97:243-251.

45. Tai FWD, McAlindon ME (2021) Non-steroidal anti-inflammatory drugs and the gastrointestinal tract. Clin Med (Lond). 21:131-134. doi: 10.7861/clinmed.2021-0039.

46. Varga Z, Sabzwari SRA, Vargova V (2017) Cardiovascular Risk of Nonsteroidal Anti-Inflammatory Drugs: An Under-Recognized Public Health Issue. Cureus. 9:e1144. doi: 10.7759/cureus.1144.

47. Drożdżal S, Lechowicz K, Szostak B, Rosik J, Kotfis K, Machoy-Mokrzyńska A, et al (2021) Kidney damage from nonsteroidal anti-inflammatory drugs—Myth or truth? Review of selected literature. Pharmacology Research & Perspectives. 9:e00817. doi: https://doi.org/10.1002/prp2.817.

48. Moreno-Ajona D, Pérez-Rodríguez A, Goadsby PJ (2020) Small-molecule CGRP receptor antagonists: A new approach to the acute and preventive treatment of migraine. Medicine in Drug Discovery. 7:100053. doi: https://doi.org/10.1016/j.medidd.2020.100053.

49. Croop R, Lipton RB, Kudrow D, Stock DA, Kamen L, Conway CM, et al (2021) Oral rimegepant for preventive treatment of migraine: a phase 2/3, randomised, double-blind, placebo-controlled trial. Lancet. 397:51-60. doi: 10.1016/s0140-6736(20)32544-7.

50. Mohajer A, Scripture J, L'Italien G, Harris L, Coric V, Rosen N (2022) Reduction in Opioid Prescription Fills and Morphine Milligram Equivalent Dispensed Following Initiation of Nurtec ODT Treatment – A Real World Administrative Claims Study (S31.010). Neurology. 98:3807.

51. Rosen N, Mohajer A, Abraham L, Brown J, Hygge Blakeman K, Jenkins A, et al (2023) Reduction in barbiturate prescription fills and quantity dispensed following initiation of rimegepant treatment: A real-world administrative claims study. Headache. 63:78.

52. L'Italien G, Harris L, Mohajer A, Scripture J, Coric V, Rosen N (2022) Real world evidence of reduction in point prevalence of medication overuse headache after migraine therapy with rimegepant. Headache. 62:80.

53. Vandenbussche N, Laterza D, Lisicki M, Lloyd J, Lupi C, Tischler H, et al (2018) Medication-overuse headache: a widely recognized entity amidst ongoing debate. The Journal of Headache and Pain. 19:50. doi: 10.1186/s10194-018-0875-x.

54. Ashina S, Terwindt GM, Steiner TJ, Lee MJ, Porreca F, Tassorelli C, et al (2023) Medication overuse headache. Nat Rev Dis Primers. 9:5. doi: 10.1038/s41572-022-00415-0.

55. Saengjaroentham C, Strother LC, Dripps I, Sultan Jabir MR, Pradhan A, Goadsby PJ, et al (2020) Differential medication overuse risk of novel anti-migraine therapeutics. Brain. 143:2681-2688. doi: 10.1093/brain/awaa211.

56. Navratilova E, Behravesh S, Oyarzo J, Dodick DW, Banerjee P, Porreca F (2020) Ubrogepant does not induce latent sensitization in a preclinical model of medication overuse headache. Cephalalgia. 40:892-902. doi: 10.1177/0333102420938652.

图例

图 1. 研究设计

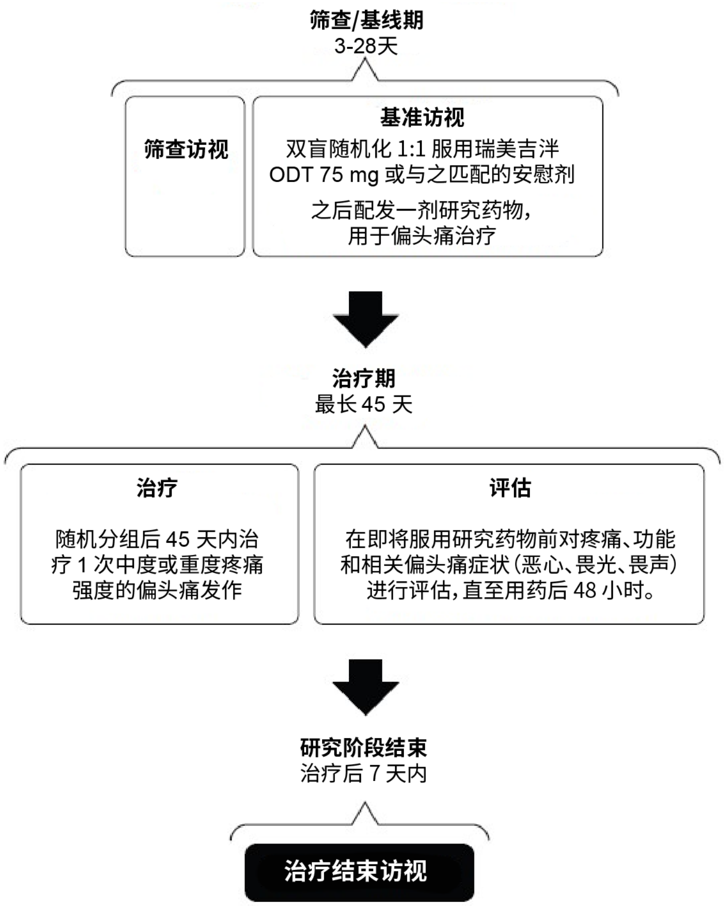

ODT，口腔崩解片。

图 2. 从服药后 15 分钟到 48 小时，无痛、无 MBS、疼痛缓解和功能恢复正常的受试者比例的变化。

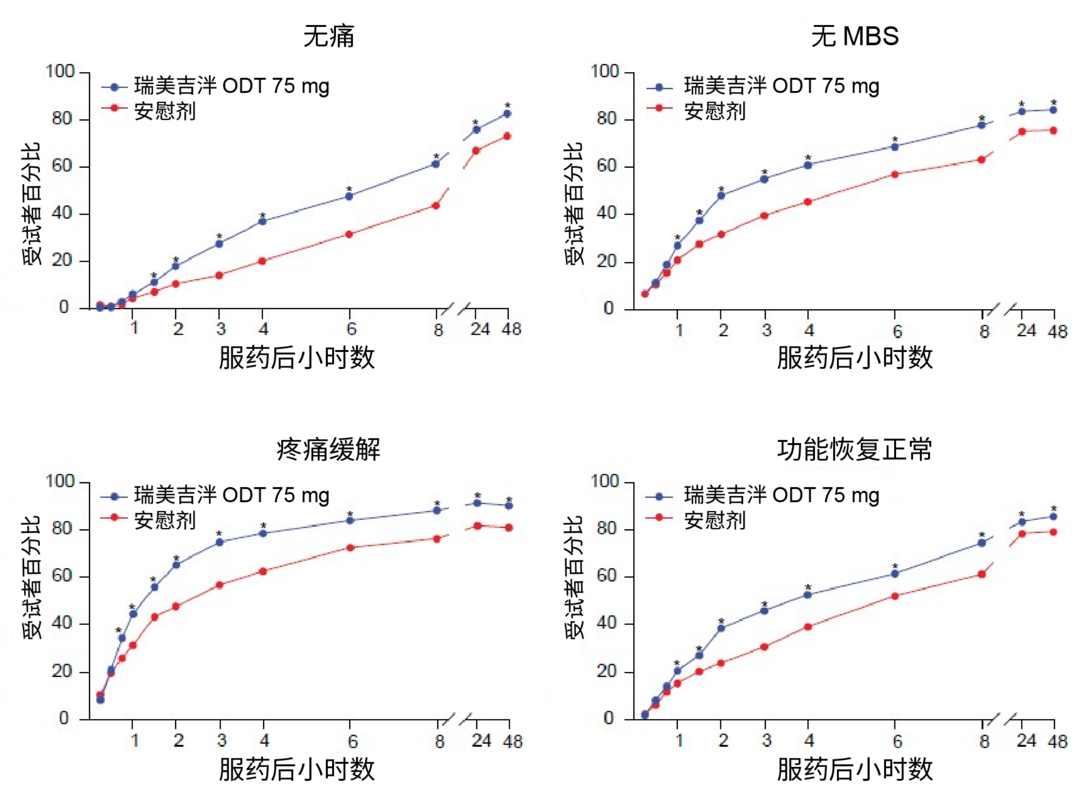

在给药后 15 分钟、30 分钟、45 分钟、1 小时、1.5 小时、2 小时、3 小时、4 小时、6 小时、8 小时、24 小时和 48 小时进行评估。在评估时间点数据缺失或在评估时间点或之前使用过挽救药物的受试者被归为试验失败（在偏头痛发病时未报告 MBS 的受试者，在无 MBS 分析中归为失败）。

*名义 *p* 值<0.05；基于风险差异（瑞美吉泮 ODT 与安慰剂对比）。

MBS，最令人困扰的症状； ODT，口腔崩解片。

表 1. 所有接受研究治疗的中国受试者的人口统计学和临床特征

**人口统计学**	**瑞美吉泮 75 毫克**<br>***n* =538**	**安慰剂**<br>***n* =537**	**总计**<br>***N* = 1075**
年龄，岁			
平均值（标准差）	37.3 (10.3)	36.7 (10.4)	37.3 (10.3)
中位数（范围）	36 (19–71)	35 (18–70)	35 (18–71)
性别，n (%)			
女性	412 (76.6)	437 (81.4)	849 (79.0)
男性	126 (23.4)	100 (18.6)	226 (21.0)
体重指数[a]， kg/m^2			
平均值（标准差）	22.9 (3.4)	23.1 (3.4)	23.0 (3.4)
中位数（范围）	22.5 (15.6–47.0)	22.8 (15.5–35.8)	22.7 (15.5–47.0)
原发性偏头痛类型，n (%)			
无先兆	476 (88.5)	476 (88.6)	952 (88.6)
有先兆	62 (11.5)	61 (11.4)	123 (11.4)
发病年龄，岁[b]			
平均值（标准差）	27.1 (9.2)	26.2 (8.6)	26.7 (8.9)
中位数（范围）	27 (3–49)	26 (6–49)	26 (3–49)
未经治疗发作的平均持续时间，小时			
平均值（标准差）	18.0 (14.7)	18.4 (14.7)	18.2 (14.6)
中位数（范围）	12 (4–72)	12 (4–72)	12 (4–72)
每月中度或重度疼痛强度的发作次数			
平均值（标准差）	3.5 (1.3)	3.4 (1.2)	3.5 (1.2)
中位数（范围）	3 (2–8)	3 (2–8)	3 (2–8)
曾经最令人困扰的症状，n (%)			
恶心	281 (52.2)	278 (51.8)	559 (52.0)
畏声	155 (28.8)	157 (29.2)	312 (29.0)
畏光	102 (19.0)	101 (18.8)	203 (18.9)
缺失	0	1 (0.2)	1 (0.1)
之前接受过预防性偏头痛治疗，n (%)	21 (3.9)	17 (3.2)	38 (3.5)

[a] 受试者数量 = 瑞美吉泮 537，安慰剂 537，总计 1074。
[b] 受试者数量 = 瑞美吉泮 532，安慰剂 532，总计 1064。
SD，标准差。

表 2. 共同主要终点和关键次要终点[a]的总结

治疗终点	瑞美吉泮 75 毫克 *n* =537	安慰剂 *n* =537	风险差异[b] (95% 置信区间)	*p* 值[c]
**共同主要终点**				
给药后 2 小时无痛	98 (18.2%)	57 (10.6%)	7.6 (3.5, 11.8)	0.0004
给药后 2 小时无 MBS	258 (48.0%)	171 (31.8%)	16.2 (10.4, 22.0)	< 0.0001
**关键次要终点**				
给药后 2 小时疼痛缓解	351 (65.4%)	256 (47.7%)	17.8 (12.0, 23.7)	< 0.0001
给药后 2 小时功能恢复正常[d]	176 (38.5%)	110 (23.8%)	14.7 (8.8, 20.6)	< 0.0001
给药后 24 小时内使用挽救药物	28 (5.2%)	75 (14.0%)	−8.9 (−12.4, −5.4)	< 0.0001
给药后 2-24 小时持续无痛	82 (15.3%)	43 (8.0%)	7.2 (3.4, 11.1)	0.0002
给药后 2-48 小时持续无痛	78 (14.5%)	39 (7.3%)	7.2 (3.5, 10.9)	0.0001

[a] 包括所有接受研究治疗、治疗时偏头痛程度为中度或重度、并提供至少一个治疗后疗效数据点的随机中国受试者。有关处理缺失数据和失败定义的详细信息，请参阅研究方法部分。

[b] 瑞美吉泮与安慰剂对比，根据预防性偏头痛药物使用情况分层进行 Mantel–Haenszel 检验计算得出。

[c] 瑞美吉泮与安慰剂对比，根据预防性偏头痛药物使用情况分层进行 Cochran-Mantel-Haenszel 检验计算得出。所有 *p* 值均为名义值。

[d] 在给药时有功能障碍的受试者中（瑞美吉泮， $n = 457$；安慰剂， $n = 462$）。

MBS，最令人困扰的症状。

表 3. 所有接受治疗的中国受试者的治疗期间不良事件概要

**TEAE，n (%)**	**瑞美吉泮 75 毫克**<br>***n*** **=538**	**安慰剂**<br>***n*** **=537**
任何 TEAE	82 (15.2)	88 (16.4)
严重 TEAE	1 (0.2)[a]	2 (0.4)
最常见的 TEAE[b]		
尿蛋白阳性	8 (1.5)	7 (1.3)
恶心	6 (1.1)	14 (2.6)
尿路感染	5 (0.9)	8 (1.5)
血肌酸磷酸激酶升高	5 (0.9)	3 (0.6)
蛋白尿	4 (0.7)	1 (0.2)
畏光	4 (0.7)	3 (0.6)
上呼吸道感染	3 (0.6)	4 (0.7)
尿潜血	3 (0.6)	2 (0.4)
血糖升高	3 (0.6)	1 (0.2)
尿白细胞酯酶阳性	3 (0.6)	1 (0.2)
**研究药物相关**		
任何 TEAE	45 (8.4)	43 (8.0)
严重 TEAE	0	1 (0.2)

[a] 该严重 TEAE 使用 MedDRA（23.0 版）首选术语“感染”进行分类。

[b] 瑞美吉泮 ODT 75 毫克组有≥ 0.5%的受试者出现 TEAE。

MedDRA，ODT，口腔崩解片； TEAE，治疗期间不良事件。

补充材料

补充表 1. 从服药后 15 分钟到 48 小时，无痛、 无 MBS 、疼痛缓解和功能恢复正常的受试者比例的变化。[a]

治疗终点	瑞美吉泮 75 毫克 *n* =537	安慰剂 *n* =537	风险差异[b] (95% 置信区间)	*p* 值[c]
**无痛**				
给药后 15 分钟	4 (0.7%)	9 (1.7%)	-0.9 (-2.2, 0.4)	0.1683
给药后 30 分钟	5 (0.9%)	6 (1.1)	-0.2 (-1.4, 1.0)	0.7739
给药后 45 分钟	17 (3.2%)	11 (2.0%)	1.1 (-0.8, 3.0)	0.2708
给药后 60 分钟	32 (6.0%)	24 (4.5%)	1.5 (-1.1, 4.2)	0.2567
给药后 90 分钟	61 (11.4%)	39 (7.3%)	4.1 (0.7, 7.6)	0.0204
给药后 2 小时	98 (18.2%)	57 (10.6%)	7.6 (3.5, 11.8)	0.0004
给药后 3 小时	149 (27.7%)	77 (14.3%)	13.3 (8.5, 18.2)	< 0.0001
给药后 4 小时	199 (37.1%)	109 (20.3%)	16.7 (11.4, 22.0)	< 0.0001
给药后 6 小时	257 (47.9%)	170 (31.7%)	16.2 (10.4, 22.0)	< 0.0001
给药后 8 小时	331 (61.6%)	235 (43.8%)	18.0 (12.1, 23.8)	< 0.0001
给药后 24 小时	408 (76.0%)	360 (67.0%)	9.2 (3.8, 14.6)	0.0008
给药后 48 小时	445 (82.9%)	394 (73.4%)	9.6 (4.7, 14.5)	0.0001
**无 MBS**				
给药后 15 分钟	35 (6.5%)	36 (6.7%)	-0.1 (-3.1, 2.9)	0.9349
给药后 30 分钟	62 (11.5%)	57 (10.6%)	0.9 (-2.9, 4.6)	0.6419
给药后 45 分钟	102 (19.0%)	83 (15.5%)	3.5 (-1.0, 8.0)	0.1306
给药后 60 分钟	145 (27.0%)	112 (20.9%)	6.2 (1.1, 11.3)	0.0168
给药后 90 分钟	202 (37.6%)	148 (27.6%)	10.0 (4.4, 15.6)	0.0005
给药后 2 小时	258 (48.0%)	171 (31.8%)	16.2 (10.4, 22.0)	< 0.0001
给药后 3 小时	296 (55.1%)	213 (39.7%)	15.6 (9.7, 21.5)	< 0.0001
给药后 4 小时	328 (61.1%)	244 (45.4%)	15.7 (9.8, 21.6)	< 0.0001
给药后 6 小时	370 (68.9%)	306 (57.0%)	12.0 (6.3, 17.8)	< 0.0001
给药后 8 小时	418 (77.8%)	340 (63.3%)	14.7 (9.3, 20.1)	< 0.0001
给药后 24 小时	449 (83.6%)	404 (75.2%)	8.6 (3.8, 13.4)	0.0005
给药后 48 小时	453 (84.4%)	406 (75.6%)	8.9 (4.2, 13.7)	0.0003
**疼痛缓解**				
给药后 15 分钟	44 (8.2%)	56 (10.4%)	-2.2 (-5.7, 1.3)	0.2108
给药后 30 分钟	112 (20.9%)	106 (19.7%)	1.1 (-3.7, 5.9)	0.6566
给药后 45 分钟	184 (34.3%)	139 (25.9%)	8.4 (2.9, 13.9)	0.0028

给药后 60 分钟	239 (44.5%)	167 (31.1%)	13.6 (7.8, 19.3)	< 0.0001
给药后 90 分钟	300 (55.9%)	233 (43.4%)	12.5 (6.6, 18.5)	< 0.0001
给药后 2 小时	351 (65.4%)	256 (47.7%)	17.8 (12.0, 23.7)	< 0.0001
给药后 3 小时	402 (74.9%)	305 (56.8%)	18.1 (12.5, 23.6)	< 0.0001
给药后 4 小时	422 (78.6%)	336 (62.6%)	16.0 (10.6, 21.4)	< 0.0001
给药后 6 小时	451 (84.0%)	390 (72.6%)	11.3 (6.4, 16.2)	< 0.0001
给药后 8 小时	474 (88.3%)	410 (76.4%)	12.1 (7.6, 16.6)	< 0.0001
给药后 24 小时	491 (91.4%)	440 (81.9%)	9.7 (5.7, 13.7)	< 0.0001
给药后 48 小时	485 (90.3%)	435 (81.0%)	9.4 (5.3, 13.6)	< 0.0001
**功能恢复正常** [d]				
给药后 15 分钟	8 (1.8%)	11 (2.4%)	-0.6 (-2.5, 1.2)	0.5116
给药后 30 分钟	37 (8.1%)	28 (6.1%)	2.1 (-1.2, 5.4)	0.2181
给药后 45 分钟	64 (14.0%)	54 (11.7%)	2.2 (-2.1, 6.6)	0.3145
给药后 60 分钟	94 (20.6%)	71 (15.4%)	5.2 (0.2, 10.2)	0.0402
给药后 90 分钟	124 (27.1%)	94 (20.3%)	6.8 (1.3, 12.3)	0.0160
给药后 2 小时	176 (38.5%)	110 (23.8%)	14.7 (8.8, 20.6)	< 0.0001
给药后 3 小时	210 (46.0%)	142 (30.7%)	15.2 (9.0, 21.5)	< 0.0001
给药后 4 小时	241 (52.7%)	181 (39.2%)	13.6 (7.2, 20.0)	< 0.0001
给药后 6 小时	281 (61.5%)	241 (52.2%)	9.3 (3.0, 15.7)	0.0043
给药后 8 小时	341 (74.6%)	283 (61.3%)	13.5 (7.6, 19.5)	< 0.0001
给药后 24 小时	382 (83.6%)	363 (78.6%)	5.1 (0.1, 10.2)	0.0469
给药后 48 小时	392 (85.8%)	367 (79.4%)	6.5 (1.6, 11.3)	0.0097

[a] 包括所有接受研究治疗、治疗时偏头痛程度为中度或重度、并提供至少一个治疗后疗效数据点的随机中国受试者。有关处理缺失数据和失败定义的详细信息，请参阅研究方法部分。

[b] 瑞美吉泮与安慰剂对比，根据预防性偏头痛药物使用情况分层进行 Mantel–Haenszel 检验计算得出。

[c] 瑞美吉泮与安慰剂对比，根据预防性偏头痛药物使用情况分层进行 Cochran-Mantel-Haenszel 检验计算得出。所有 p 值均为名义值。

[d] 在给药时有功能障碍的受试者中（瑞美吉泮， $n = 457$；安慰剂， $n = 462$）。

MBS，最令人困扰的症状。

512
